# Analyzing and Quantifying the Gain-of-Function Enhancement of IP_3_ Receptor Gating by Familial Alzheimer’s Disease-Causing Mutants in Presenilins

**DOI:** 10.1371/journal.pcbi.1004529

**Published:** 2015-10-06

**Authors:** Don-On Daniel Mak, King-Ho Cheung, Patrick Toglia, J. Kevin Foskett, Ghanim Ullah

**Affiliations:** 1 Department of Physiology, University of Pennsylvania, Philadelphia, Pennsylvania, United States of America; 2 Department of Physiology, The University of Hong Kong, Pok Fu Lam, Hong Kong; 3 Department of Physics, University of South Florida, Tampa, Florida, United States of America; 4 Department of Cell and Developmental Biology, University of Pennsylvania, Philadelphia, Pennsylvania, United States of America; The Krasnow Institute for Advanced Studies, UNITED STATES

## Abstract

Familial Alzheimer’s disease (FAD)-causing mutant presenilins (PS) interact with inositol 1,4,5-trisphosphate (IP_3_) receptor (IP_3_R) Ca^2+^ release channels resulting in enhanced IP_3_R channel gating in an amyloid beta (A*β*) production-independent manner. This gain-of-function enhancement of IP_3_R activity is considered to be the main reason behind the upregulation of intracellular Ca^2+^ signaling in the presence of optimal and suboptimal stimuli and spontaneous Ca^2+^ signals observed in cells expressing mutant PS. In this paper, we employed computational modeling of single IP_3_R channel activity records obtained under optimal Ca^2+^ and multiple IP_3_ concentrations to gain deeper insights into the enhancement of IP_3_R function. We found that in addition to the high occupancy of the high-activity (H) mode and the low occupancy of the low-activity (L) mode, IP_3_R in FAD-causing mutant PS-expressing cells exhibits significantly longer mean life-time for the H mode and shorter life-time for the L mode, leading to shorter mean close-time and hence high open probability of the channel in comparison to IP_3_R in cells expressing wild-type PS. The model is then used to extrapolate the behavior of the channel to a wide range of IP_3_ and Ca^2+^ concentrations and quantify the sensitivity of IP_3_R to its two ligands. We show that the gain-of-function enhancement is sensitive to both IP_3_ and Ca^2+^ and that very small amount of IP_3_ is required to stimulate IP_3_R channels in the presence of FAD-causing mutant PS to the same level of activity as channels in control cells stimulated by significantly higher IP_3_ concentrations. We further demonstrate with simulations that the relatively longer time spent by IP_3_R in the H mode leads to the observed higher frequency of local Ca^2+^ signals, which can account for the more frequent global Ca^2+^ signals observed, while the enhanced activity of the channel at extremely low ligand concentrations will lead to spontaneous Ca^2+^ signals in cells expressing FAD-causing mutant PS.

## Author Summary

Aberrant Ca^2+^ signaling caused by IP_3_R gating dysregulation is implicated in many neurodegenerative diseases such as Alzheimer’s, Huntington’s, Spinocerebellar ataxias, and endoplasmic reticulum stress-induced brain damage. Thus understanding IP_3_R dysfunction is important for the etiology of these diseases. It was previously shown that FAD-causing mutant PS interacts with the IP_3_R, leading to its gain-of-function enhancement in optimal Ca^2+^ and sub-saturating IP_3_ concentrations. Here, we use data-driven modeling to provide deeper insights into the upregulation of IP_3_R gating in a wide range of ligand concentrations and quantify the sensitivity of the channel to its ligands in the presence of mutant PS. Our simulations demonstrate that these changes can alter the statistics of local Ca^2+^ events and we speculate that they lead to Ca^2+^ signaling dysregulations at the whole cell level observed in FAD cells. These models will provide the foundation for future data-driven computational framework for local and global Ca^2+^ signals that will be used to judiciously isolate the primary pathways causing Ca^2+^ dysregulation in FAD from those that are downstream, and to study the effects of upregulation of IP_3_R activity on cell function.

## Introduction

Alzheimer’s disease (AD) is a fatal neurodegenerative disease that leads to cognitive, memory, and behavioral impairments followed by progressive cell death. The symptoms of AD include the extracellular deposition of amyloid *β* (A*β*) plaques and intracellular neurofibrillary tangles—aggregates of microtubule-associated protein *τ* [1]. According to the amyloid hypothesis, the accumulation of A*β* oligomers or plaques due to the imbalance between synthesis and clearance of A*β* is the driving force for AD pathogenesis [2]. However, whether *τ* and A*β* aggregates are the causes or symptoms of AD remains a matter of debate [3].

A*β* is produced by the cleavage of amyloid precursor protein (APP), an integral membrane protein. APP is cleaved sequentially by *β* and *γ*-secretases to generate A*β* monomers that are released to the extracellular space and form oligomers. The *γ*-secretase complex contains four different proteins including presenilins (PS) that are synthesized and localized in the ER [1]. Mutations in PS alter the APP processing, thus leading to A*β* oligomarization either through higher production or relatively higher proportion of amyloidogenic A*β* types [4]. It is well established that mutations in PS and APP are the main causes of Familial AD (FAD) [5]. What is not clear is how PS mutations and A*β* accumulation lead to the impairment of brain function and neurodegeneration. The Ca^2+^ hypothesis of AD, which is based on the enhanced intracellular Ca^2+^ signaling during AD, accounts for early memory loss and subsequent cell death [6, 7, 8].

There is strong evidence in favor of intracellular Ca^2+^ signal exaggeration by FAD-causing PS mutations as an early phenotype that could contribute to the pathogenesis of the disease [9]. The exaggerated cytosolic Ca^2+^ signals are ascribed mainly to the enhanced release of Ca^2+^ from intracellular endoplasmic reticulum (ER) store due to overloading of ER lumen by up-regulated sarco-endoplasmic reticulum Ca^2+^-ATPase (SERCA) pump [10]; disruption of ER-membrane Ca^2+^ leak channels [11]; or enhanced gating of IP_3_R [12, 13, 14, 15], the ubiquitous ER-localized Ca^2+^ release channel crucial for the generation and modulation of intracellular Ca^2+^ signals in animal cells [16]. Single channel studies in multiple cell lines show that the sensitivity of IP_3_R to its agonist IP_3_ increases significantly in the presence of FAD-causing mutant PS [14, 15], leading to a several fold increase in the open probability (*P*_o_) of IP_3_R channel in subsaturating IP_3_. These studies were performed in the absence of A*β*, suggesting that the modulation of IP_3_R is a major mechanism for intracellular Ca^2+^ signal dysregulation in cells expressing FAD-causing mutant PS. Furthermore, altered IP_3_R-mediated Ca^2+^ release has been suggested as the fundamental defect and highly predictive diagnostic feature in AD [17]. Suppression of IP_3_R-mediated Ca^2+^ signaling was recently shown to restore normal cell function and memory tasks in M146V (FAD-causing PS mutation) knock-in [18] and triple-transgenic [19] mice models of FAD [20].

IP_3_R gates in three distinct modes: an “L” mode with very low *P*_o_, in which brief openings are separated by quiescent periods with long mean closed channel durations (*τ*_c_); an “I” mode with intermediate *P*_o_, in which the channel opens and closes rapidly with short *τ*_c_ and mean open channel durations (*τ*_o_); and an “H” mode with high *P*_o_, in which the channel gates in bursts [21]. All three modes are observed under all conditions in which the channel gates, and the channel spontaneously switches among all three modes even under constant ligand conditions. The *P*_o_ of the channel remains remarkably consistent in each gating mode in all ligand conditions so the ligand dependencies of overall channel *P*_o_, *τ*_o_ and *τ*_c_ come from the ligand dependencies of the relative prevalence (normalized occupancy) *π* ^M^ of the gating modes (M can be L, I, and H) [21].

Due to the significant role of IP_3_R-mediated Ca^2+^ signaling dysregulation in AD, a comprehensive understanding of the IP_3_R function is important for both the etiology of the disease and designing effective therapeutic reagents. It was discovered that IP_3_R channels in cells expressing FAD-causing mutant PS exhibit relatively higher *π* ^H^ and lower *π* ^L^ in comparison to IP_3_R in cells expressing wild-type PS [15, 14] in non-optimal ligand conditions. *π* ^I^, on the other hand, remains largely the same. The switch in the prevalence of H and L modes causes the increase in *P*_o_ of IP_3_R in the presence of FAD-causing mutant PS.

In this paper, we employ a data-driven modeling approach to gain further insights into the gating behavior of IP_3_R in the presence of wild-type and FAD-causing PS. We focus on the channel gating behaviors of endogenous IP_3_R in the presence of human wild-type (PS1-WT) and FAD-causing mutant (PS1-M146L) PS expressed in the Sf9 cells, an insect cell line derived from the moth Spodoptera frugiperda. Other FAD-causing mutant PS1 (PS1-L116P, PS1-G384A) and PS2 (PS2-N141I) have similar effects on IP_3_R channel gating as PS1-M146L. On the other hand, non-FAD-associated mutant PS1 (PS1-L113P and PS1-G183V), wild-type PS2 and EVER1 (an irrelevant ER transmembrane protein) have little to no effects on IP_3_R channel gating, like PS1-WT [15, 14]. Therefore, the conclusions from studying IP_3_R channel in the presence of PS1-WT (IP_3_R_PS1WT_) and IP_3_R channel in the presence of PS1-M146L (IP_3_R_PS1M146L_) can be generalized to other FAD-causing mutations as well. We used the data-driven kinetic model developed to describe *all* observed gating behaviors of the endogenous IP_3_R channels in Sf9 cells: channel *P*_o_, *τ*_o_ and *τ*_c_ distributions in various steady Ca^2+^ and IP_3_ concentrations (𝓒 and 𝓘, respectively); modal gating behaviors in various steady 𝓒 and 𝓘; and kinetic response of IP_3_R channels to abrupt changes in 𝓘 and/or 𝓒 [22] as the starting point of our approach. By modifying a minimum number of model parameters, the data-driven model was applied to fit observed gating behaviors: channel *P*_o_, *τ*_o_, *τ*_c_, and modal prevalence, in optimal (1 *μ*M) 𝓒 and sub-saturating (100 nM) 𝓘, of the Sf9 IP_3_R_PS1M146L_ and IP_3_R_PS1WT_ (used as control) [15] as well as *P*_o_, *τ*_o_, and *τ*_c_ of IP_3_R_PS1WT_ in 33 nM and 10*μ*M 𝓘 at 𝓒 = 1*μ*M. In addition to elucidating the kinetics and factors contributing to the gain-of-function enhancement of IP_3_R activity [14, 15], we extrapolate, using our modified model, the gating behavior of IP_3_R_PS1WT_ and IP_3_R_PS1M146L_ for a wide range of 𝓒 and 𝓘. We also quantify and compare the ligand sensitivities of IP_3_R_PS1M146L_ and IP_3_R_PS1WT_. Simulations of local Ca^2+^-release events based on the results of the data-driven model demonstrate that the gain-of-function enhancement of IP_3_R activity leads to larger, longer, and more frequent local Ca^2+^ releases events in cells expressing FAD-causing PS mutants. The models derived here will provide the foundation for developing future data-driven computational framework for global intracellular Ca^2+^ signals that will be used to judiciously isolate the primary factors causing Ca^2+^ signaling dysregulation in FAD from those that are downstream, and to study the effects of upregulation of IP_3_R activity on cell functions such as ATP production.

## Materials and Methods

### Experimental Methods

The main experimental data used in this paper for fitting the models were previously published elsewhere [15]. Basic experimental data (*P*_o_, *τ*_o_, and *τ*_c_) at 𝓘 = 33nM, and 10*μ*M for both IP_3_R_PS1WT_ and IP_3_R_PS1M146L_ [14] were also used to generate our model. The full details of experimental methods are given in [15] and summarized below.

Two Sf9 cell lines expressing recombinant PS1-WT and PS1-M146L, respectively, were generated and maintained as described in [14].

Nuclei isolated from transfected Sf9 cells [23, 14] were used for nuclear patch clamp experiments in on-nucleus configuration at room temperature [24]. All experimental solutions contained 140 mM KCl and 10 mM HEPES (pH 7.3). Bath solution contained 90 nM free Ca^2+^ (buffered by 0.5 mM BAPTA (1,2-bis(2-aminophenoxy)ethane-N,N,N’,N’-tetraacetic acid). Pipette solution contained 1 *μ*M free Ca^2+^ (buffered by 0.5 mM 5, 5′-dibromo-BAPTA), 0.5 mM Na_2ATP_ and sub-saturating 100 nM IP_3_.

Segments of current records exhibiting current levels for a single IP_3_R channel were idealized with QuB software (University of Buffalo) using SKM algorithm [25, 26]. The idealized current traces were further analyzed as described in [21] to characterized the modal gating behaviors of IP_3_R channels. Short closing events, presumably caused by ligand-independent transitions [27], were removed by burst analysis. Gating modes were assigned according to durations of channel burst (T_*b*_) and burst-terminating gaps (T_*g*_) [21], using a critical T_*b*_ of 100 ms and a critical T_*g*_ of 200 ms to detect modal transitions.

### Computational Methods

**Fig 1:**
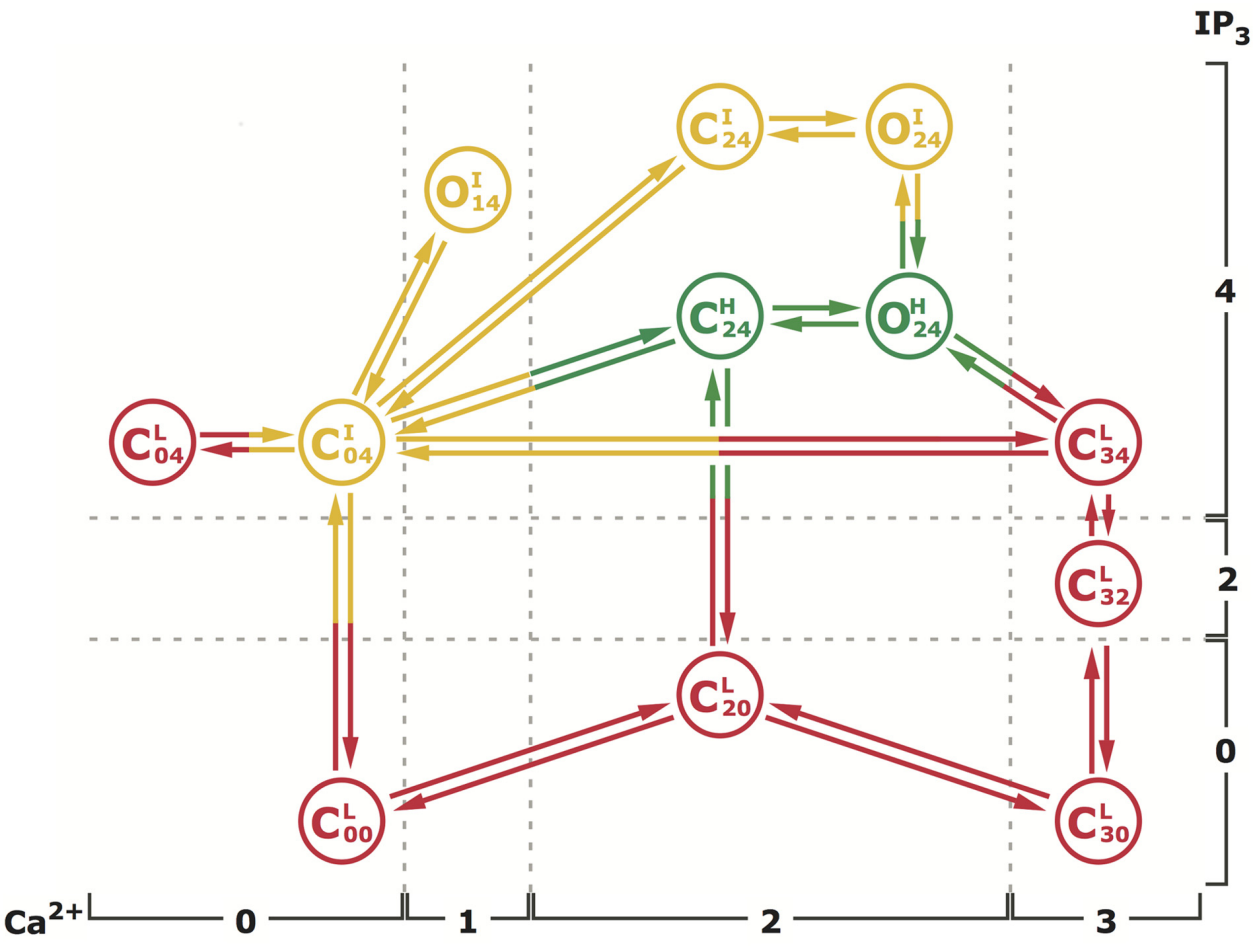
The kinetic scheme for the proposed model. The superscript of each state denote the gating mode that state is in. The two numbers in the subscripts respectively indicate the number of Ca^2+^ and IP_3_ bound to the channel in that state. The model has nine closed states: ${\text{C}}_{00}^{\text{L}}$, ${\text{C}}_{20}^{\text{L}}$, ${\text{C}}_{30}^{\text{L}}$, ${\text{C}}_{32}^{\text{L}}$, ${\text{C}}_{34}^{\text{L}}$, ${\text{C}}_{04}^{\text{L}}$, ${\text{C}}_{04}^{\text{I}}$, ${\text{C}}_{24}^{\text{I}}$, and ${\text{C}}_{24}^{\text{H}}$ and three open states: ${\text{O}}_{14}^{\text{I}}$, ${\text{O}}_{24}^{\text{I}}$, and ${\text{O}}_{24}^{\text{H}}$. The transition rates between various states and the related flux parameters are listed in Tables 2 and 3 respectively.

We fit the twelve-state model previously developed for IP_3_R in Sf9 cells [22] (Fig 1) to the channel gating data of Sf9 IP_3_R_PS1WT_ and IP_3_R_PS1M146L_ at 𝓒 = 1*μ*M and 𝓘 = 100 nM [15], following the procedure in [22, 28, 29], which we describe below. Although the scheme in Fig 1 seems to suggest simultaneous binding/unbinding of multiple ligands as the channel goes from one state to another (for instance ${\text{C}}_{00}^{L}$ to ${\text{C}}_{20}^{L}$, or ${\text{C}}_{24}^{H}$ to ${\text{C}}_{20}^{L}$), in reality, there is no such direct transition in our model. Each one of such transitions actually involves one or more intermediate states that are not explicitly shown in order to keep the scheme simple. As discussed in detail in [22], the simplifying approximations were made by considering the fact that the intermediate states have relatively low occupancy and therefore can be aggregated into the main states. The rates for the composite transitions between those main states explicitly shown in the scheme were actually derived with the intermediate low-occupancy states carefully taken into consideration.

#### Occupancy parameters

First, we derive the optimal parameters specifying the occupancy of various gating states from the mean *P*_o_ and prevalences of the three modes.

*Open Probability*: In the following, ${\text{C}}_{\text{mn}}^{\text{M}}$ and ${\text{O}}_{\text{mn}}^{\text{M}}$ represent a closed and open state respectively in the M mode (where M = L, I, or H) with m Ca^2+^ and n IP_3_ bound to the channel. Relative to the reference unliganded closed state ${\text{C}}_{00}^{\text{L}}$, the occupancies of ${\text{C}}_{\text{mn}}^{\text{M}}$ and ${\text{O}}_{\text{mn}}^{\text{M}}$ states are proportional to 𝓒^*m*^𝓘^*n*^, with occupancy parameters $K_{{\text{C}}_{mn}^{\text{M}}}$ and $K_{{\text{O}}_{mn}^{\text{M}}}$, respectively. The occupancy parameter of a state is equal to the product of equilibrium association constants along any path connecting ${\text{C}}_{00}^{L}$ to that state ($K_{{\text{C}}_{00}^{\text{L}}}=1$). The normalized occupancy of C_*mn*_ and O_*mn*_ are $K_{{\text{C}}_{mn}^{\text{M}}}{𝓒}^{m}{𝓘}^{n}/Z$ and $K_{{\text{O}}_{mn}^{\text{M}}}{𝓒}^{m}{𝓘}^{n}/Z$, respectively, where *Z*, the total occupancy of all states, is given by

$$\begin{array}{ccc} Z & = & \left\{Z_{o}\right\}+\left\{Z_{c}\right\}=\left\{K_{O_{14}^{I}}𝒞{ℐ}^{4}+K_{O_{24}^{I}}{𝒞}^{2}{ℐ}^{4}+K_{O_{24}^{H}}{𝒞}^{2}{ℐ}^{4}\right\}\\ & & +\;\{K_{C_{00}^{L}}+K_{C_{20}^{L}}{𝒞}^{2}+K_{C_{30}^{L}}{𝒞}^{3}+K_{C_{32}^{L}}{𝒞}^{3}{ℐ}^{2}+K_{C_{34}^{L}}{𝒞}^{3}{ℐ}^{4}\\ & & +\;K_{C_{04}^{L}}{ℐ}^{4}+K_{C_{04}^{I}}{ℐ}^{4}+K_{C_{24}^{I}}{𝒞}^{2}{ℐ}^{4}+K_{C_{24}^{H}}{𝒞}^{2}{ℐ}^{4}\;\}. \end{array}$$ (1)

The equilibrium *P*_o_ function is:

$$\begin{array}{c} P_{o}\left(𝒞,ℐ\right)=\frac{Z_{o}}{Z}\;. \end{array}$$ (2)

*Modal Prevelances*: The IP_3_R channel exhibits three distinct gating modes [21]. The relative prevalence, *π* ^M^, of the gating mode [21] is given as

$$\begin{array}{c} \pi^{M}=Z^{M}/Z, \end{array}$$ (3)

where *Z* ^M^ is the sum of occupancies of all states in a given mode. For example, for H mode, *Z* ^H^ = $K_{{\text{C}}_{24}^{\text{H}}}{𝓒}^{2}{𝓘}^{4}+K_{{\text{O}}_{24}^{\text{H}}}{𝓒}^{2}{𝓘}^{4}$.

We fit *P*_o_ *π* ^L^, *π* ^I^, and *π* ^H^ function simultaneously to the *P*_o_ and prevalence data using the Mathematica routine “NonlinearModelFit” based on least squares fitting. We perform this fit for single channel data from both IP_3_R_PS1WT_ and IP_3_R_PS1M146L_ to get the optimal occupancy parameters for IP_3_R in the presence of wild-type and FAD-causing mutant PS.

#### Probability flux parameters

To determine the parameters describing the probability flux between different model states [22] involved in the transition rates between various states, we performed maximum likelihood fits on current traces of channel gating from IP_3_R_PS1WT_ and IP_3_R_PS1M146L_.

*Maximum Likelihood and AIC Calculations*: We calculated the “log-likelihood” function for the model in the open-source programming language “Octave” and used the function “nelder_mead_min” in the optimization tool kit to minimize the likelihood score (−*log*(*likelihood*(*data*))) of the time-series data by varying the 18 free flux parameters while holding the occupancy parameters fixed. The log of the likelihood function for the current traces is given as [30]

$$\begin{array}{ccc} log(likelihood\left(tc_{1},to_{1},tc_{2},to_{2},\ldots tc_{n},to_{n}\right)) & = & log(\pi_{C}exp\left(Q_{CC}tc_{1}\right)Q_{CO}exp\left(Q_{OO}to_{1}\right)\\ & & Q_{OC}exp\left(Q_{CC}tc_{2}\right)Q_{CO}exp\left(Q_{OO}to_{2}\right)\ldots \\ & & exp\left(Q_{OO}to_{n}\right)u_{O}), \end{array}$$ (4)

where *π*_*C*_ is the initial probability of closed states being occupied at equilibrium, *to*_*i*_ and *tc*_*i*_ are the ith opening and closing in the time-series respectively, and *Q*_*CC*_, *Q*_*OO*_, *Q*_*OC*_, and *Q*_*CO*_ are the sub-matrices of the 12 × 12 generator matrix *Q*. That is,

$$\begin{array}{c} Q=(\begin{array}{cc} Q_{OO} & Q_{OC}\\ Q_{CO} & Q_{CC} \end{array}). \end{array}$$ (5)

The element of *Q* at location ij, *Q*_ij_,i ≠ j is the transition rate from state i to state j. The diagonal entries are given by *Q*_ii_ = −∑_j ≠ i_ *Q*_ij_, which is an expression of conservation of probability [31]. Thus *Q*_*CC*_, *Q*_*OO*_, *Q*_*OC*_, and *Q*_*CO*_ are matrices of the transition rates from all closed to all closed, all open to all open, all open to all closed, and all closed to all open states, respectively. Since our model has 9 closed and 3 open states, *Q*_*CC*_, *Q*_*OO*_, *Q*_*CO*_, and *Q*_*OC*_ are 9 × 9, 3 × 3, 9 × 3, and 3 × 9 matrices respectively. For data obtained at equilibrium, *π*_*C*_ = *W*_*O*_ *Q*_*OC*_/*J*, with *J* = *W*_*O*_ *Q*_*OC*_ *u*_*C*_, where *u*_*C*_ is a nine—(the number of close states) component vector of all 1’s. *W*_*C*_ and *W*_*O*_ are diagonal matrices of the equilibrium occupancies of all closed and all open states respectively.

The total log-likelihood of all data used in the fit was calculated as

$$\begin{array}{c} log\left(likelihood\left(data\right)\right)=\sum_{i=1}^{N_{exp}}log\left(likelihood\left(data_{i}\right)\right), \end{array}$$ (6)

where *N*_*exp*_ is the number of experiments and *data*_*i*_ is the data set (time series) from experiment *i*. A total of 30 and 15 experiments were used in the global fit for the IP_3_R gating in the presence of PS1-WT and PS1-M146L, respectively.

#### Mean open and closed times

The mean open and closed times are given by

$$\begin{array}{ccc} \tau_{o} & = & P_{o}\left(𝒞,ℐ\right)/J\\ \tau_{c} & = & (1-P_{o}\left(𝒞,ℐ\right))/J, \end{array}$$ (7)

where *J* is the total equilibrium flux from the 3 open states to the closed states. The equilibrium flux from a given state to other states is the product of the occupancy of that state and the sum of transition rates from that state to others. Thus, *J* is given as [22]:

$$\begin{array}{ccc} J & = & K_{O_{24}^{H}}{𝒞}^{2}{ℐ}^{4}\times \left(r_{\left(O_{24}^{H}\to \;C_{24}^{H}\right)}+r_{\left(O_{24}^{H}\to \;C_{34}^{L}\right)}\right)\\ & & +\;K_{O_{24}^{I}}{𝒞}^{2}{ℐ}^{4}\times r_{\left(O_{14}^{I}\to \;C_{24}^{I}\right)}\\ & & +\;K_{O_{14}^{I}}𝒞{ℐ}^{4}\times r_{\left(O_{14}^{I}\to \;C_{04}^{I}\right)}, \end{array}$$ (8)

where *r*_(*S* → *U*)_ is the transition rate from state S to state U.

#### Mean modal lifetimes

The lifetime *τ* ^X^ of any aggregate X (mode or other combinations of Markovian states), in an aggregated Markov chain is given by

$$\begin{array}{c} \tau^{X}=\frac{Z^{X}}{J_{X}}, \end{array}$$ (9)

where *Z* ^X^ is the unnormalized occupancy of aggregate X and *J*_X_ the unnormalized flux out of that aggregate. *J*_X_ is the sum of all the fluxes from all reactions from all Markovian states contained in the aggregate X to Markovian states not contained in X, so *J*_X_ = ∑_S_ *J*_S_ = ∑_S_(*Z*_S_∑_U_ *r*_(S → U)_), where ∑_S_ is summing over all Markovian states S in the aggregate X, ∑_U_ is summing over all Markovian states U that are *not* in X. For example for H mode, $\tau^{\text{H}}=\frac{Z^{\text{H}}}{J_{\text{H}}}$, where $Z^{\text{H}}=K_{{\text{C}}_{24}^{\text{H}}}{𝓒}^{2}{𝓘}^{4}+K_{{\text{O}}_{24}^{\text{H}}}{𝓒}^{2}{𝓘}^{4}$ and $J_{\text{H}}=K_{{\text{O}}_{24}^{\text{H}}}{𝓒}^{2}{𝓘}^{4}(r_{\left({\text{O}}_{24}^{\text{H}}\to {\text{C}}_{34}^{\text{L}}\right)}+r_{\left({\text{O}}_{24}^{\text{H}}\to {\text{O}}_{24}^{\text{I}}\right)})+K_{{\text{C}}_{24}^{\text{H}}}{𝓒}^{2}{𝓘}^{4}(r_{\left({\text{C}}_{24}^{\text{H}}\to {\text{C}}_{04}^{\text{I}}\right)}+r_{\left({\text{C}}_{24}^{\text{H}}\to {\text{C}}_{20}^{\text{L}}\right)})$.

#### Dwell-time distributions

In the following, we first derive the expressions for the open and closed dwell-time distributions and later generalize them for the dwell-time distributions in any aggregate of states. We define a 12 × 12 diagonal matrix *W* with *W*_*ii*_ equal to the equilibrium occupancy of ith state. We partition the *W* matrix into *W*_*C*_ and *W*_*O*_, where *W*_*C*_ and *W*_*O*_ are diagonal matrices of the equilibrium occupancies of the 9 closed states and 3 open states respectively in the model. The open time distribution is the probability density for a channel that opened at time 0 to close for the first time at *t*_*O*_. The probability that the channel first closed at time *t*_*O*_ is given by

$$\begin{array}{c} \frac{dp_{O}}{dt}=p_{O}Q_{OO.} \end{array}$$ (10)

which has solution *p*_*o*_(*t*_*o*_) = *π*_*O*_ *exp*(*Q*_*OO*_ *t*_*O*_). The probability, *F*_*O*_, that the channel remains open at time *t*_*O*_ is the sum of the probability over all the open states and is given as

$$\begin{array}{c} F_{O}\left(t_{O}\right)=\pi_{O}exp\left(Q_{OO}t_{O}\right)u_{O}, \end{array}$$ (11)

where *u*_*O*_ and *u*_*C*_ are column vectors of all ones having dimensions equal to the number of open and close states respectively. The probability, *G*_*C*_, that the channel closes for the first time at time *t*_*O*_ is *G*_*C*_(*t*_*O*_) = 1−*F*_*O*_(*t*_*O*_). The open dwell-time distribution, *f*_*O*_(*t*_*O*_) is defined by [32, 33, 30, 31]:

$$\begin{array}{c} \int_{0}^{t_{O}}f_{O}\left(t\right)dt=G_{C}(t_{O}), \end{array}$$ (12)

or *f*_*O*_(*t*_*O*_) = *dG*_*C*_(*t*_*O*_)/*dt*_*O*_ so that

$$\begin{array}{c} f_{O}\left(t_{O}\right)=-\pi_{O}exp\left(Q_{OO}t_{O}\right)Q_{OO}u_{O}, \end{array}$$ (13)

which can be written as

$$\begin{array}{c} f_{O}\left(t_{O}\right)=\pi_{O}exp\left(Q_{OO}t_{O}\right)Q_{OC}u_{C}. \end{array}$$ (14)

Similarly, the closed time distribution is given as

$$\begin{array}{c} f_{C}\left(t_{C}\right)=\pi_{C}exp\left(Q_{CC}t_{C}\right)Q_{CO}u_{O}. \end{array}$$ (15)

The initial probabilities of open and closed states being occupied at equilibrium are given as

$$\begin{array}{c} \pi_{O}=\frac{W_{C}Q_{CO}}{J} \end{array}$$ (16)

$$\begin{array}{c} \pi_{C}=\frac{W_{O}Q_{OC,}}{J} \end{array}$$ (17)

where *J* = *W*_*C*_ *Q*_*CO*_ *u*_*O*_ = *W*_*O*_ *Q*_*OC*_ *u*_*C*_ is the total flux from all open states to all closed states at equilibrium and vice versa.

Generalizing this result, the dwell-time distributions of aggregates *X* and *Y* respectively are given as

$$\begin{array}{c} f_{X}\left(t_{X}\right)=\pi_{X}exp\left(Q_{XX}t_{X}\right)Q_{XY}u_{Y}. \end{array}$$ (18)

Similarly, the closed time distribution is given as

$$\begin{array}{c} f_{Y}\left(t_{Y}\right)=\pi_{Y}exp\left(Q_{YY}t_{Y}\right)Q_{YX}u_{X}. \end{array}$$ (19)

The initial probabilities of states in *X* and *Y* being occupied at equilibrium are given as

$$\begin{array}{c} \pi_{X}=\frac{W_{Y}Q_{YX}}{J} \end{array}$$ (20)

$$\begin{array}{c} \pi_{Y}=\frac{W_{X}Q_{XY,}}{J} \end{array}$$ (21)

where *J* = *W*_*Y*_ *Q*_*YX*_ *u*_*X*_ = *W*_*X*_ *Q*_*XY*_ *u*_*Y*_.

For the open and close dwell-time distributions in a given mode, *X* and *Y* consist of all open and close states in the mode respectively. Thus, $X=[{\text{O}}_{24}^{\text{I}},{\text{O}}_{14}^{\text{I}}]$, $Y=[{\text{C}}_{24}^{\text{I}},{\text{C}}_{04}^{\text{I}}]$ for I mode and $X=[{\text{O}}_{24}^{\text{H}}]$, $Y=[{\text{C}}_{24}^{\text{H}}]$ for H mode. *W*_*Y*_ and *W*_*X*_ are diagonal matrices of the equilibrium occupancies of all closed states and all open states in the given mode respectively. *u*_*X*_ and *u*_*Y*_ are column vectors of all ones having dimensions equal to the number of open and close states respectively in the mode. The square matrix *Q* has the dimensions of the number of states in the mode (4 for I mode, 2 for H mode). Sub-matrices *Q*_*XY*_ of *Q* has the transition rates from all states in *X* to all states in *Y* aggregate in the mode etc. For example for I mode,

$$\begin{array}{c} Q_{XX}=(\begin{array}{cc} -r_{\left(O_{14}^{I}\to \;C_{04}^{I}\right)} & 0\\ 0 & -r_{\left(O_{24}^{I}\to \;C_{24}^{H}\right)} \end{array}) \end{array}$$ (22)

$$\begin{array}{c} Q_{XY}=(\begin{array}{cc} r_{\left(O_{14}^{I}\to \;C_{04}^{I}\right)} & 0\\ 0 & r_{\left(O_{24}^{I}\to \;C_{24}^{I}\right)} \end{array}) \end{array}$$ (23)

$$\begin{array}{c} Q_{YX}=(\begin{array}{cc} r_{\left(C_{04}^{I}\to \;O_{14}^{I}\right)} & 0\\ 0 & r_{\left(C_{24}^{I}\to \;O_{24}^{I}\right)} \end{array}) \end{array}$$ (24)

$$\begin{array}{c} Q_{YY}=(\begin{array}{cc} -r_{\left(C_{04}^{I}\to \;O_{14}^{I}\right)}-r_{\left(C_{04}^{I}\to \;C_{24}^{I}\right)} & r_{\left(C_{04}^{I}\to \;C_{24}^{I}\right)}\\ r_{\left(C_{24}^{I}\to \;C_{04}^{I}\right)} & -r_{\left(C_{24}^{I}\to \;O_{24}^{I}\right)}-r_{\left(C_{24}^{I}\to \;C_{04}^{I}\right)} \end{array}) \end{array}$$ (25)

Similar matrices can be written for H mode where each sub-matrix is of dimension one (one close and one open state each in *X* and *Y*).

#### Stochastic simulations of an IP_3_R cluster

To simulate Ca^2+^ puffs and blips (local Ca^2+^ release events from the ER due to nearly simultaneous opening of multiple channels and a single channel, respectively, in a cluster of several IP_3_R channels), we followed a procedure developed in [28] and consider a cluster of ten IP_3_R channels arranged in a two dimensional array with an inter-channel spacing of 120 *nm*. The gating of each channel is given by the twelve-state model shown in Fig 1.

Ca^2+^ concentration on the cytoplasmic side of the cluster is controlled by diffusion; the flux coming out from the ER through IP_3_R channels, *J*_*j*_; and the concentration of free dye, *b*_*d*_. Thus the rate equations for the concentrations of free Ca^2+^ *c* ^*j*^(*r*_*j*_, *t*) and free Ca^2+^ dye buffer $b_{d}^{j}(r_{j},t)$ at distance *r*_*j*_ from channel *j* and time *t* are described as below:

$$\begin{array}{ccc} \frac{\partial c^{j}\left(r_{j},t\right)}{\partial t} & = & D_{c}\nabla_{j}^{2}c_{j}+J_{j}\delta \left(r_{j}\right)+k_{d}^{r}\left(B_{d}-b_{d}^{j}\right)-k_{d}^{f}c^{j}b_{d}^{j} \end{array}$$ (26)

$$\begin{array}{ccc} \frac{\partial b_{d}^{j}}{\partial t} & = & D_{d}\nabla_{j}^{2}b_{d}^{j}+k_{d}^{r}\left(B_{d}-b_{d}^{j}\right)-k_{d}^{f}c^{j}b_{d}^{j}. \end{array}$$ (27)

In the above equations, *B*_*d*_ is the total concentration, $k_{d}^{f}$ the forward (binding) rate, and $k_{d}^{r}$ the reverse (unbinding) rate for dye buffer. *D*_*c*_ and *D*_*d*_ are the diffusion coefficients for Ca^2+^ and dye respectively. *δ*(*r*_*j*_) is the Dirac delta function and *J*_*j*_ is the Ca^2+^ flux through the *j* ^*th*^ channel.

$$\begin{array}{c} J_{j}=\{\begin{array}{cc} \frac{I}{2\times F\times \delta V} & \text{for}\;r\leq \Delta r,\\ 0 & \text{for}\;r>0. \end{array} \end{array}$$ (28)

Where *I* = 0.05 *pA* is the channel current, *F* is the Faraday’s constant, Δ*r* = 2.5*nm*, and *δV* is the volume of the hemisphere over the channel having a radius of *r*_*pore*_ [34]. We assume that the Ca^2+^ pump and leak currents are slow on the time scales considered here and therefore have negligible effects. Various parameters used in Eqs (26) and (27) are given in S1 Text.

The propagation of Ca^2+^ and dye is simulated throughout a 3D cytosolic space. Considering the spherical symmetry around the channel, the Laplacian of Ca^2+^ and buffers in spherical coordinates is given as

$$\begin{array}{c} \nabla_{j}^{2}X\left(r_{j},t\right)=\frac{1}{r_{j}^{2}}\frac{\partial }{\partial r_{j}}(r_{j}^{2}\frac{\partial X}{\partial r_{j}}) \end{array}$$ (29)

where $X=c^{j},\text{or}b_{d}^{j}$.

We solved the two differential equations Eqs (26) and (27) using an implicit numerical method based on finite differences for discretizing the system of PDEs on a hemispherical volume of radius 5 *μm* with a spatial grid size of 5 *nm* for each channel as described in the S1 Text and summed the contribution of all channels for the instantaneous Ca^2+^ concentration at a given point in space. The Ca^2+^ concentration at the location of each channel is updated by adding the contributions from other channels in the cluster

$$\begin{array}{c} {𝒞}_{i}=\sum_{j=1}^{10}c^{j}\left(r_{ij}\right) \end{array}$$ (30)

Where *r*_*ji*_ is the distance between channels *i* and *j*.

## Results

**Fig 2:**
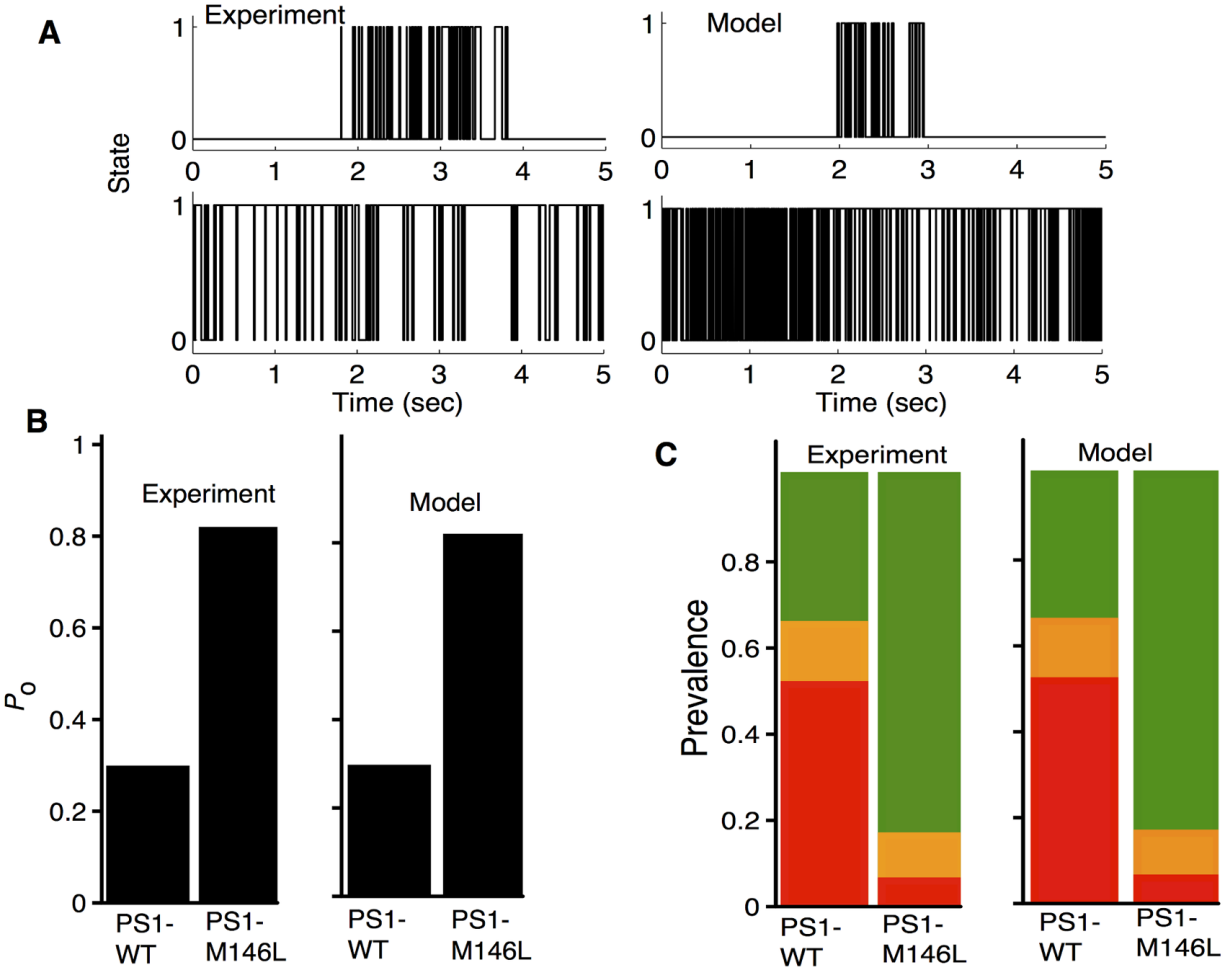
Effect of PS1-WT and PS1-M146L on the gating of IP_3_R. (A) Representative idealized experimental (left) and model-simulated (right) time-traces for IP_3_R_PS1WT_ (top) and IP_3_R_PS1M146L_ (bottom) at 𝓒 = 1*μ*M and 𝓘 = 100nM, where 0 and 1 represent closed and open states, respectively. (B and C) Experimental (left) and model-simulated (right) *P*_o_ and *π* ^M^, respectively, of IP_3_R channels in the presence of PS1-WT and PS1-M146L. In (C), relative prevalence of the L, I, and H modes are in red, orange and green, respectively.

**Fig 3:**
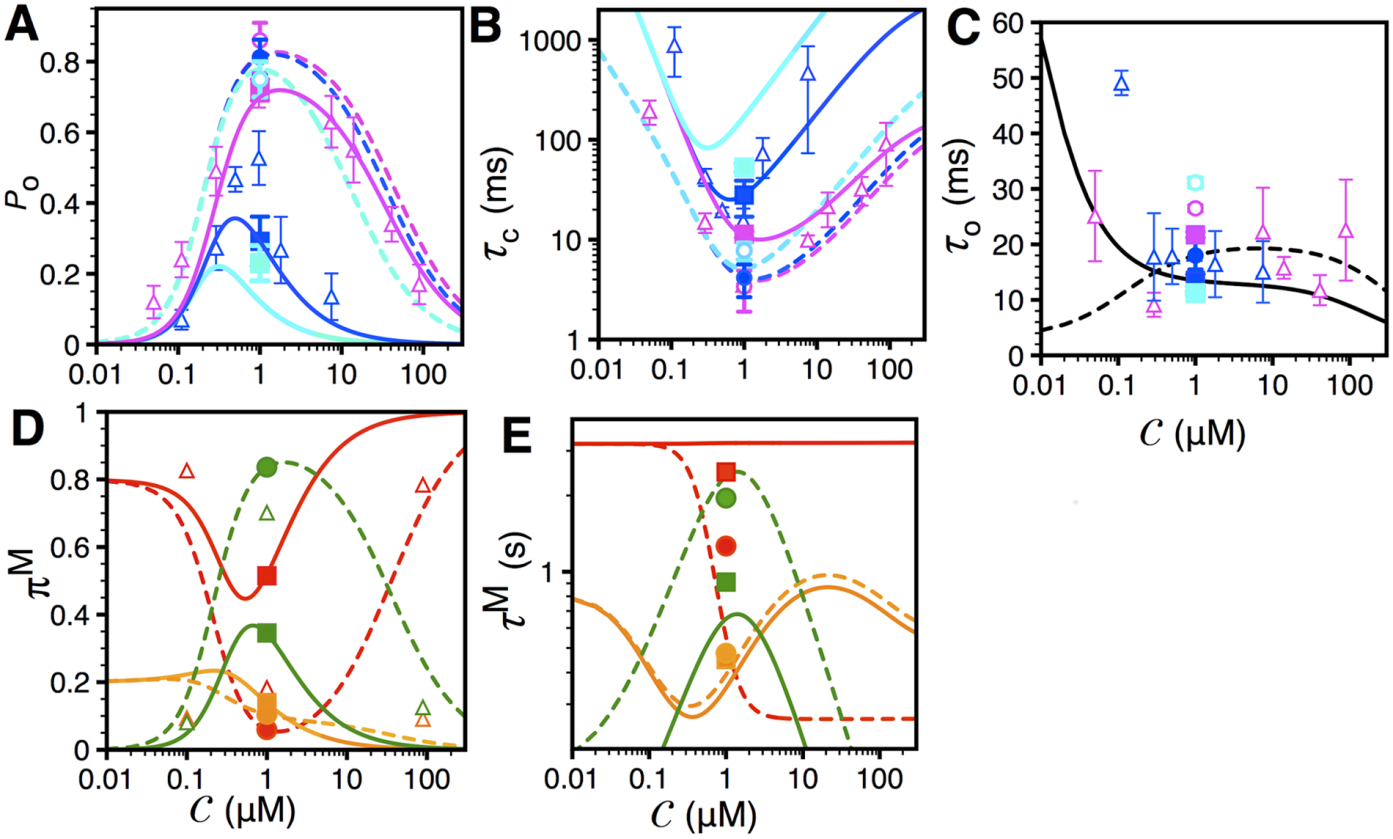
Mean gating properties of IP_3_R in the presence of PS1-WT and PS1-M146L. Experimental values are shown by symbols (filled symbols for data that are used to derive the parameters for our model, open symbols for data not used for parameter derivation), theoretical values calculated from the twelve-state model using corresponding parameters are shown by lines. Mean *P*_o_ (A), *τ*_c_ (B), and *τ*_o_ (C) of IP_3_R_PS1WT_ (solid lines and squares) and IP_3_R_PS1M146L_ (dashed lines and circles) at 𝓘 = 33nM, 100nM, and 10*μ*M (in turquoise, blue, and magenta, respectively) as functions of 𝓒. Data for 𝓘 = 100nM (blue symbols) were published in [15], and data for 𝓘 = 33 nM and 10 uM (turquoise and magenta symbols, respectively) were published in [14]. (C) Because experimental *τ*_o_ values did not show any strong systematic trend as 𝓘 varied (from 33 nM through 100 nM to 10 *μ*M), theoretical values of *τ*_o_ generated by the models for all 𝓘 (33nM, 100 nM or 10 *μ*M) are the same (shown in black, solid line for IP_3_R_PS1WT_ and dashed line for IP_3_R_PS1M146L_). Prevalences (D) and life-times (E) of the three gating modes (red, orange, and green for L, I, and H modes, respectively) of IP_3_R_PS1WT_ (solid lines and squares) and IP_3_R_PS1M146L_ (dashed lines and circles) as a function of 𝓒 at 𝓘 = 100nM. In (D), the open triangles are data for IP_3_R_noPS1_ at saturating 𝓘 = 10*μ*M [21] showing that modal prevalences of IP_3_R_PS1M146L_ at 𝓘 = 100 nM are similar to those of IP_3_R_noPS1_ at 𝓘 = 10*μ*M. Error bars represent standard error of the mean.

Representative time-series traces of the gating behavior of IP_3_R_PS1WT_ and IP_3_R_PS1M146L_ are shown in Fig 2A. Occupancy parameters for the twelve states in the model obtained by fitting the *P*_o_ (Figs 2B, 3A) and prevalence (Fig 2C) data from IP_3_R_PS1WT_ and IP_3_R_PS1M146L_ are given in Table 1. Notice that some of the parameters for IP_3_R_PS1WT_ are different from those in [22] because IP_3_R_PS1WT_ behaves somewhat differently from the IP_3_R channel in wild type untransfected Sf9 cells (IP_3_R_noPS1_), despite the general similarity in the gating of the two (Fig 3A–3C, triangles for IP_3_R_noPS1_, squares and solid lines for IP_3_R_PS1WT_), especially *P*_o_ at 𝓘 = 100nM and 400 nM < 𝓒 ≤ 1*μ*M. It is remarkable that the occupancy of only 4 states changes in the presence of PS1-M146L as compared to PS1-WT. The four states are $K_{{\text{O}}_{24}^{\text{H}}},K_{{\text{O}}_{24}^{\text{I}}},K_{{\text{O}}_{14}^{\text{I}}}$, and $K_{{\text{C}}_{32}^{\text{L}}}$ whose occupancies change by a factor of 4.587, 1.284, 1.718, and 0.018 respectively. Thus IP_3_R_PS1M146L_ spends relatively more time in the states $K_{{\text{O}}_{24}^{\text{H}}},K_{{\text{O}}_{24}^{\text{I}}}$, and $K_{{\text{O}}_{14}^{\text{I}}}$ and less time in $K_{{\text{C}}_{32}^{\text{L}}}$ as compared to IP_3_R_PS1WT_. This is consistent with the prevalence data where there is a significant increase in *π* ^H^ (0.345 vs 0.836) at the cost of *π* ^L^ (0.515 vs 0.059) in IP_3_R_PS1M146L_ as compared to IP_3_R_PS1WT_. *π* ^I^ on the other hand does not change significantly (0.14 vs 0.104) (Fig 2C). Thus the increase in *P*_o_ is mainly due to the significantly less time spent by IP_3_R_PS1M146L_ in ${\text{C}}_{32}^{\text{L}}$ and more time spent in ${\text{O}}_{24}^{\text{H}}$ (Fig 2B) as compared to IP_3_R_PS1WT_.

**Table 1:** Parameters for occupancies of all states in the model. Parameters for IP_3_R_PS1M146L_ are shown in bold if they are different from those for IP_3_R_PS1WT_. All other parameters are the same for IP_3_R_PS1WT_ and IP_3_R_PS1M146L_.

Parameters	Values
$K_{{\text{C}}_{00}^{\text{L}}}$	1
$K_{{\text{C}}_{20}^{\text{L}}}$	7.061 *μ*M^−2^
$K_{{\text{C}}_{30}^{\text{L}}}$	1.778 *μ*M^−3^
$K_{{\text{C}}_{32}^{\text{L}}}$	1.504 × 10^7^ **(2.749 × 10^5^)** *μ*M^−5^
$K_{{\text{C}}_{04}^{\text{L}}}$	1.746 × 10^8^ *μ*M^−4^
$K_{{\text{C}}_{04}^{\text{I}}}$	4.365 × 10^7^ *μ*M^−4^
$K_{{\text{C}}_{24}^{\text{H}}}$	3.082 × 10^8^ *μ*M^−6^
$K_{{\text{C}}_{24}^{\text{I}}}$	3.0823 × 10^8^ *μ*M^−4^
$K_{{\text{C}}_{34}^{\text{L}}}$	1.0319 × 10^8^ *μ*M^−7^
$K_{{\text{O}}_{14}^{\text{I}}}$	6.605 × 10^7^ **(8.478 × 10^7^)** *μ*M^−5^
$K_{{\text{O}}_{24}^{\text{H}}}$	8.702 × 10^8^ **(3.992 × 10^9^)** *μ*M^−6^
$K_{{\text{O}}_{24}^{\text{I}}}$	5.856 × 10^7^ **(1.006 × 10^8^)** ***μ*M** ^−6^

To derive the probability flux parameters used in the transition rates between different gating states (Table 2), we fit the model to idealized current traces recording openings and closings of IP_3_R_PS1WT_ and IP_3_R_PS1M146L_ by minimizing the likelihood score Eq (6) of the data. The flux parameters from the fits are given in Table 3. Only two of the eighteen flux parameters of IP_3_R_PS1M146L_ are different from IP_3_R_PS1WT_ (shown in bold). These two parameters are involved in the ${\text{C}}_{04}^{\text{I}}↔{\text{O}}_{14}^{\text{I}}$ and ${\text{C}}_{24}^{\text{H}}↔{\text{O}}_{24}^{\text{H}}$ transitions and are higher for IP_3_R_PS1M146L_ as compared to IP_3_R_PS1WT_.

**Table 2:** Transition rates between various states. In the following *j*_*ijmn*_ represents the flux parameter (see Table 3) and *rsr* stands for reciprocal of sum of reciprocals, i.e. *rsr*(*x*, *y*) = 1/(1/*x*+1/*y*). For simplicity, we assume that the rates for the ${\text{C}}_{04}^{\text{I}}\to {\text{C}}_{24}^{\text{I}}$ & ${\text{C}}_{00}^{\text{L}}\to {\text{C}}_{20}^{\text{L}}$ transitions are equal to the rate for ${\text{C}}_{04}^{\text{I}}\to {\text{C}}_{24}^{\text{H}}$ transition while those for ${\text{C}}_{04}^{\text{I}}\leftarrow {\text{C}}_{24}^{\text{I}}$ & ${\text{C}}_{00}^{\text{L}}\leftarrow {\text{C}}_{20}^{\text{L}}$ transitions are equal to the rate for ${\text{C}}_{04}^{\text{I}}\leftarrow {\text{C}}_{24}^{\text{H}}$ transition.

Transition	Rates
${\text{C}}_{04}^{\text{I}}\to {\text{C}}_{24}^{\text{H}}$	$rsr(j_{0414}𝓒,j_{1424}{𝓒}^{2})/K_{{\text{C}}_{04}^{\text{I}}}$
${\text{C}}_{04}^{\text{I}}\leftarrow {\text{C}}_{24}^{\text{H}}$	$rsr(j_{0414}𝓒,j_{1424}{𝓒}^{2})/K_{{\text{C}}_{24}^{\text{H}}}{𝓒}^{2}$
${\text{C}}_{24}^{\text{H}}\to {\text{O}}_{24}^{\text{H}}$	$j_{2424}^{\text{HH}}/K_{{\text{C}}_{24}^{\text{H}}}$
${\text{C}}_{24}^{\text{H}}\leftarrow {\text{O}}_{24}^{\text{H}}$	$j_{2424}^{\text{HH}}/K_{{\text{O}}_{24}^{\text{H}}}$
${\text{O}}_{24}^{\text{H}}\to {\text{C}}_{34}^{\text{L}}$	$j_{2434}𝓒/K_{{\text{O}}_{24}^{\text{H}}}$
${\text{O}}_{24}^{\text{H}}\leftarrow {\text{C}}_{34}^{\text{L}}$	$j_{2434}/K_{{\text{C}}_{34}^{\text{L}}}$
${\text{C}}_{04}^{\text{I}}\to {\text{C}}_{34}^{\text{L}}$	$rsr(j_{0414}^{\text{IL}}𝓒,j_{1424}^{\text{IL}}{𝓒}^{2},j_{2434}^{\text{IL}}{𝓒}^{3})/K_{{\text{C}}_{04}^{\text{I}}}$
${\text{C}}_{04}^{\text{I}}\leftarrow {\text{C}}_{34}^{\text{L}}$	$rsr(j_{0414}^{\text{IL}}𝓒,j_{1424}^{\text{IL}}{𝓒}^{2},j_{2434}^{\text{IL}}{𝓒}^{3})/K_{{\text{C}}_{34}^{\text{L}}}{𝓒}^{3}$
${\text{C}}_{04}^{\text{I}}\to {\text{O}}_{14}^{\text{I}}$	$j_{0414}^{\text{II}}𝓒/K_{{\text{C}}_{04}^{\text{I}}}$
${\text{C}}_{04}^{\text{I}}\leftarrow {\text{O}}_{14}^{\text{I}}$	$j_{0414}^{\text{II}}/K_{{\text{O}}_{14}^{\text{I}}}$
${\text{C}}_{24}^{\text{I}}\to {\text{O}}_{24}^{\text{I}}$	$j_{2424}^{\text{II}}/K_{{\text{C}}_{24}^{\text{I}}}$
${\text{C}}_{24}^{\text{I}}\leftarrow {\text{O}}_{24}^{\text{I}}$	$j_{2424}^{\text{II}}/K_{{\text{O}}_{24}^{\text{I}}}$
${\text{O}}_{24}^{\text{I}}\to {\text{O}}_{24}^{\text{H}}$	$j_{2424}/K_{{\text{O}}_{24}^{\text{I}}}$
${\text{O}}_{24}^{\text{I}}\leftarrow {\text{O}}_{24}^{\text{H}}$	$j_{2424}/K_{{\text{O}}_{24}^{\text{H}}}$
${\text{C}}_{04}^{\text{I}}\to {\text{C}}_{04}^{\text{L}}$	$j_{0404}/K_{{\text{C}}_{04}^{\text{I}}}$
${\text{C}}_{04}^{\text{I}}\leftarrow {\text{C}}_{04}^{\text{L}}$	$j_{0404}/K_{{\text{C}}_{04}^{\text{L}}}$
${\text{C}}_{32}^{\text{L}}\to {\text{C}}_{34}^{\text{L}}$	$j_{3334}{𝓘}^{2}/K_{{\text{C}}_{32}^{\text{L}}}$
${\text{C}}_{32}^{\text{L}}\leftarrow {\text{C}}_{34}^{\text{L}}$	$j_{3334}/K_{{\text{C}}_{34}^{\text{L}}}$
${\text{C}}_{30}^{\text{L}}\to {\text{C}}_{32}^{\text{L}}$	$j_{3132}{𝓘}^{2}/K_{{\text{C}}_{30}^{\text{L}}}$
${\text{C}}_{30}^{\text{L}}\leftarrow {\text{C}}_{32}^{\text{L}}$	$j_{3132}/K_{{\text{C}}_{32}^{\text{L}}}$
${\text{C}}_{20}^{\text{L}}\to {\text{C}}_{30}^{\text{L}}$	$j_{2030}𝓒/K_{{\text{C}}_{20}^{\text{L}}}$
${\text{C}}_{20}^{\text{L}}\leftarrow {\text{C}}_{30}^{\text{L}}$	$j_{2030}/K_{{\text{C}}_{30}^{\text{L}}}$
${\text{C}}_{00}^{\text{L}}\to {\text{C}}_{04}^{\text{I}}$	*rsr*(*j*_0001_𝓘, *j*_0304_𝓘^4^)
${\text{C}}_{00}^{\text{L}}\leftarrow {\text{C}}_{04}^{\text{I}}$	$rsr(j_{0001},j_{0304}{𝓘}^{3})/K_{{\text{C}}_{04}^{\text{I}}}{𝓘}^{3}$
${\text{C}}_{20}^{\text{L}}\to {\text{C}}_{24}^{\text{H}}$	*rsr*(*j*_2021_𝓘, *j*_2324_𝓘^4^)
${\text{C}}_{20}^{\text{L}}\leftarrow {\text{C}}_{24}^{\text{H}}$	$rsr(j_{2021},j_{2324}{𝓘}^{3})/K_{{\text{C}}_{04}^{\text{I}}}{𝓘}^{3}$

**Table 3:** Flux parameters used in the model. *j*_*ijmn*_ represent a flux parameter between a state with *i* Ca^2+^ ions and *j* IP_3_ molecules and a state with *m* Ca^2+^ ions and *n* IP_3_ molecules bound. Superscripts are used to distinguish between different flux parameters that connect different pairs of states that have the same numbers of ligands bound. For example, in both transitions ${\text{C}}_{24}^{\text{I}}⇌{\text{O}}_{24}^{\text{I}}$ and ${\text{C}}_{24}^{\text{H}}⇌{\text{O}}_{24}^{\text{H}}$, the channel is bound to the same number of Ca^2+^ and IP_3_. However, the two transitions have different flux parameters because the channel is in different gating modes. The same font convention is used for the parameters as in Table 1.

Parameters	Pathway	Values	Units
*j*_0414_	${\text{C}}_{04}^{\text{I}}⇌{\text{C}}_{24}^{\text{H}}$	1.017 × 10^6^	μM^−5^ms^−1^
*j*_1424_	${\text{C}}_{04}^{\text{I}}⇌{\text{C}}_{24}^{\text{H}}$	2.840 × 10^7^	μM^−6^ms^−1^
*j*_2434_	${\text{O}}_{24}^{\text{H}}⇌{\text{C}}_{34}^{\text{L}}$	4.961 × 10^5^	μM^−7^ms^−1^
$j_{0414}^{\text{IL}}$	${\text{C}}_{04}^{\text{I}}⇌{\text{C}}_{34}^{\text{L}}$	9.502 × 10^8^	μM^−5^ms^−1^
$j_{1424}^{\text{IL}}$	${\text{C}}_{04}^{\text{I}}⇌{\text{C}}_{34}^{\text{L}}$	6.4 × 10^5^	μM^−6^ms^−1^
$j_{2434}^{\text{IL}}$	${\text{C}}_{04}^{\text{I}}⇌{\text{C}}_{34}^{\text{L}}$	2.431 × 10^3^	μM^−7^ms^−1^
*j*_2030_	${\text{C}}_{20}^{\text{L}}⇌{\text{C}}_{30}^{\text{L}}$	2.449 × 10^−3^	μM^−3^ms^−1^
$j_{0414}^{\text{II}}$	${\text{C}}_{04}^{\text{I}}⇌{\text{O}}_{14}^{\text{I}}$	5.156 × 10^5^ **(2.587 × 10^7^)**	μM^−5^ms^−1^
$j_{2424}^{\text{II}}$	${\text{C}}_{24}^{\text{I}}⇌{\text{O}}_{24}^{\text{I}}$	3.0577 × 10^7^	μM^−7^ms^−1^
*j*_2424_	${\text{O}}_{24}^{\text{I}}⇌{\text{O}}_{24}^{\text{H}}$	3.301 × 10^5^	μM^−6^ms^−1^
*j*_0404_	${\text{C}}_{04}^{\text{I}}⇌{\text{C}}_{04}^{\text{L}}$	5.459 × 10^4^	μM^−4^ms^−1^
$j_{2424}^{\text{HH}}$	${\text{C}}_{24}^{\text{H}}⇌{\text{O}}_{24}^{\text{H}}$	5.478 × 10^7^ **(1.753 × 10^8^)**	μM^−6^ms^−1^
*j*_3132_	${\text{C}}_{30}^{\text{L}}⇌{\text{C}}_{34}^{\text{L}}$	2.891 × 10^−2^	μM^−5^ms^−1^
*j*_3334_	${\text{C}}_{32}^{\text{L}}⇌{\text{C}}_{34}^{\text{L}}$	2.120 × 10^3^	μM^−7^ms^−1^
*j*_0001_	${\text{C}}_{00}^{\text{L}}⇌{\text{C}}_{04}^{\text{I}}$	1.138 × 10^−2^	μM^−1^ms^−1^
*j*_0304_	${\text{C}}_{00}^{\text{L}}⇌{\text{C}}_{04}^{\text{I}}$	4.756 × 10^10^	μM^−4^ms^−1^
*j*_2021_	${\text{C}}_{20}^{\text{L}}⇌{\text{C}}_{24}^{\text{H}}$	8.904 × 10^−4^	μM^−3^ms^−1^
*j*_2324_	${\text{C}}_{20}^{\text{L}}⇌{\text{C}}_{24}^{\text{H}}$	8.523 × 10^6^	μM^−6^ms^−1^

The mean gating properties of the channel as a function of 𝓒 at different 𝓘 values are shown in Fig 3. Theoretical values of the *P*_o_ of IP_3_R_PS1WT_ (Fig 3A, solid lines) and IP_3_R_PS1M146L_ (Fig 3A, dashed lines) were generated by using Eq (2) and the occupancy parameters in Table 1. The *P*_o_ of IP_3_R_PS1M146L_ at 𝓘 = 33nM (turquoise dashed line and turquoise circle) and 100nM (blue dashed line and blue circle) are significantly larger than *P*_o_ of IP_3_R_PS1WT_ at 𝓘 = 100nM (solid blue line and blue square). In fact, the *P*_o_ of IP_3_R_PS1M146L_ at 𝓘 = 33nM is comparable to *P*_o_ of IP_3_R_PS1WT_ (magenta square and solid line) or IP_3_R_noPS1_ (magenta triangles from [23]) at saturating 𝓘 = 10*μ*M. The *P*_o_ data for IP_3_R_noPS1_ at saturating 𝓘 = 10*μ*M is shown for comparison to emphasize that IP_3_R_PS1M146L_ is already maximally activated at a significantly lower 𝓘 of 100nM. This clearly indicates that IP_3_R in the presence of FAD-causing mutant PS is highly sensitized to activation by IP_3_, and is maximally activated at significantly lower 𝓘 than IP_3_R_PS1WT_ or IP_3_R_noPS1_. For 𝓒 > 800nM, *P*_o_ of IP_3_R_PS1M146L_ at 𝓘 = 10*μ*M (magenta dashed line and magenta circle) is higher than that of IP_3_R_noPS1_ or IP_3_R_PS1WT_ at the corresponding 𝓘 and 𝓒, mostly due to the higher saturating *P*_o_ of IP_3_R_PS1M146L_ (0.86 ± 0.03) as compared to those of IP_3_R_noPS1_ (0.72 ± 0.03) [23] and IP_3_R_PS1WT_ (0.72 ± 0.05) [15]. A close examination of *τ*_c_ (Fig 3B) and *τ*_o_ (Fig 3C) reveals that the increase in *P*_o_ of IP_3_R_PS1M146L_ is mostly due to the substantial shortening of *τ*_c_ with relatively modest increase in *τ*_o_. Theoretical values of *τ*_o_ and *τ*_c_ were calculated using Eq (7) and the parameters in Tables 1 and 3. Gating properties (*P*_o_, *τ*_o_, and *τ*_c_) of IP_3_R_noPS1_ in 𝓘 = 100nM and 10*μ*M observed in [23] (triangles in Fig 3A–3C) are similar to theoretical values calculated for IP_3_R_PS1WT_, since IP_3_R_PS1WT_ gating is generally similar to that of IP_3_R_noPS1_, as observed in [14], albeit with some noticeable differences (Fig 3A–3C). The open triangles in Fig 3A–3C representing data from [23] of IP_3_R_noPS1_ in wild type Sf9 cells is shown here to demonstrate that the model for IP_3_R_PS1WT_ can replicate reasonably well the *P*_o_, *τ*_o_, and *τ*_c_ of IP_3_R channel gating in the absence of PS1.

Next, we calculated with our modified data-driven model the prevalence of the three gating modes using Eq (3) and occupancy parameters given in Table 1 (Fig 3D and 3E). Both experimental (symbols) and theoretical (lines) results show a significant increase in *π* ^H^ (green), and decrease in *π* ^L^ (red) for IP_3_R_PS1M146L_ (dashed lines and circles) as compared to IP_3_R_PS1WT_ (solid lines and squares) (Fig 3D) at 𝓒 = 1*μ*M and 𝓘 = 100 nM. *π* ^I^ (orange), on the other hand, remains largely unchanged. Comparison with the prevalence data from IP_3_R in untransfected Sf9 cells at saturating 𝓘 = 10*μ*M (triangles) from [21] confirms the saturating activation of IP_3_R_PS1M146L_ at relatively low 𝓘. The mean life-times of the three gating modes follow a similar trend as seen in their prevalences. *τ* ^H^ is longer, *τ* ^L^ is shorter, while *τ* ^I^ remains unchanged for IP_3_R_PS1M146L_ as compared to IP_3_R_PS1WT_ (Fig 3E). The theoretical modal mean life-times were calculated from our modified model by using Eq (9) and parameters in Tables 1 and 3.

**Fig 4:**
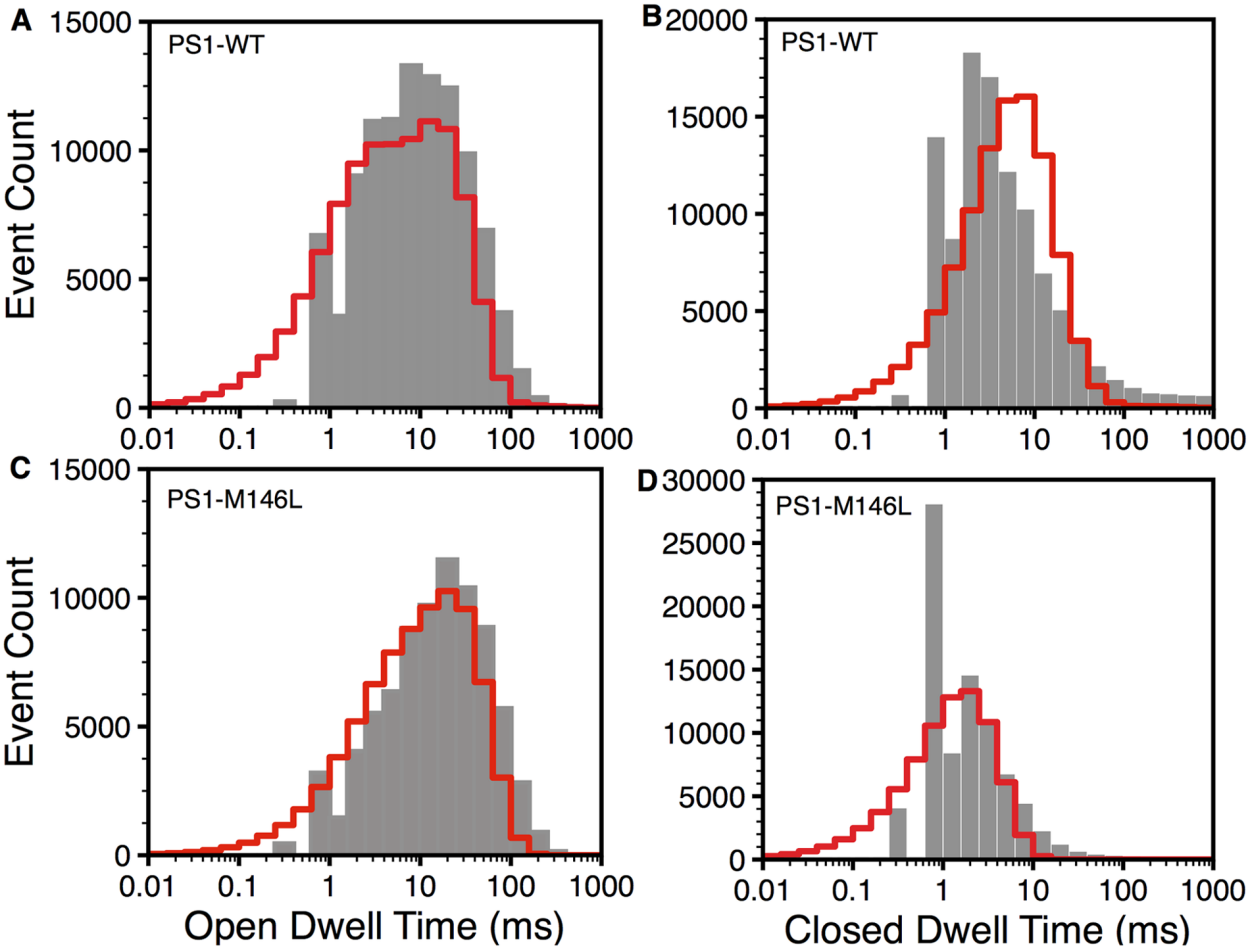
Dwell time distributions of IP_3_R. Open (A) and closed (B) dwell time distributions of IP_3_R in the presence of PS1-WT and open (C) and closed (D) dwell time distributions of IP_3_R in the presence ofPS1-M146L at 𝓒 = 1*μ*M and 𝓘 = 100nM. Bars and lines respectively represent experimental data and model.

The open and closed dwell-time distributions were calculated from the model as described in the Dwell-Time Distributions section Eqs (14) and (15) using occupancy and flux parameters in Tables 1 and 3 respectively. As shown in Fig 4, the model (red lines) fits the observed dwell-time distributions (gray bars) very well. Consistent with the *τ*_o_ observed (Fig 3C), there is a minor right-shift in the open dwell-time distribution of IP_3_R_PS1M146L_ (Fig 4C) as compared to IP_3_R_PS1WT_ (Fig 4A). Thus, PS1-M146L does not have significant effect on *τ*_o_ of the IP_3_R channel. The close dwell-time distribution of IP_3_R_PS1M146L_ (Fig 4D), on the other hand, shows significant shift to the left when compared to IP_3_R_PS1WT_ (Fig 4B), leading to the shorter *τ*_c_ and therefore the higher *P*_o_ observed.

**Fig 5:**
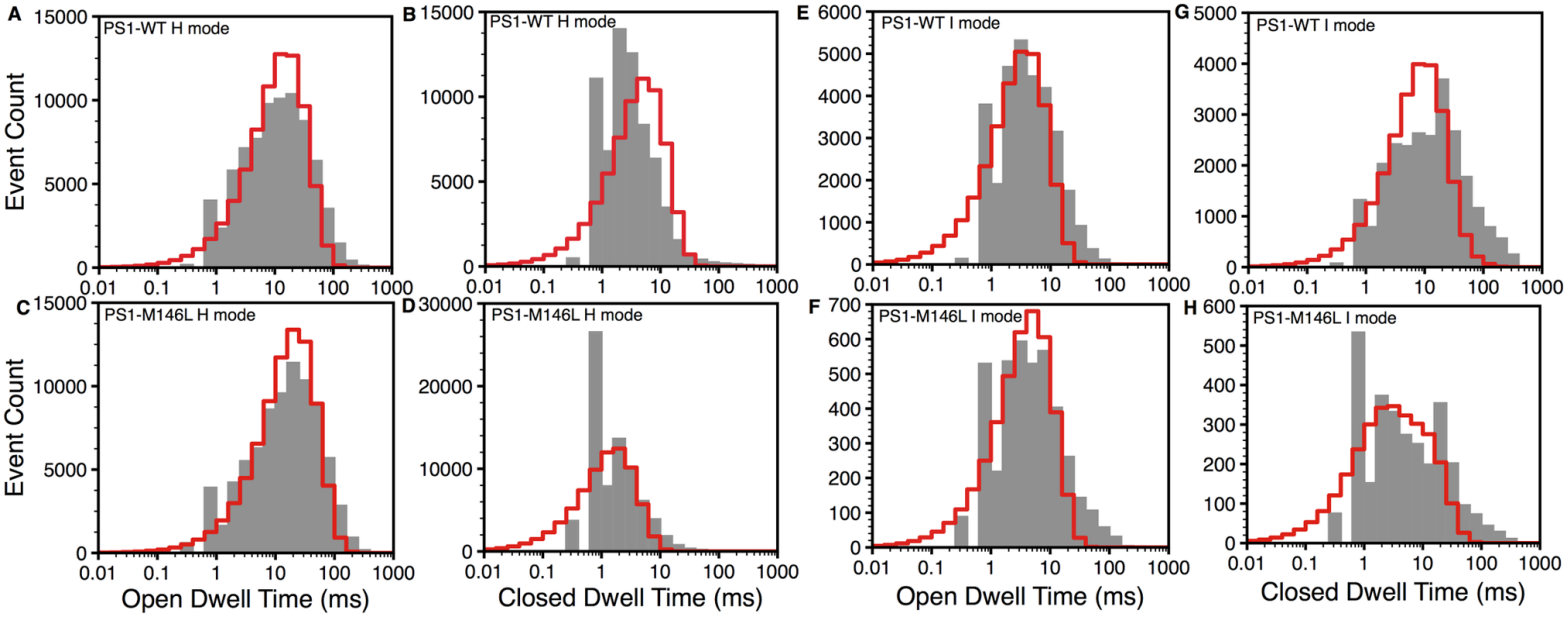
Dwell-time distributions in H and I modes at at 𝓒 = 1*μ*M and 𝓘 = 100nM. Top and bottom rows are for IP_3_R_PS1WT_ and IP_3_R_PS1M146L_ respectively. Open (A, C) and closed dwell-time distributions (B, D) in H mode. Open (E, F) and closed dwell-time distributions (G, H) in I mode. Bars are the experimental values and lines are the model fits.

The Dwell-Time Distributions section also describes the derivation of the open and closed dwell-time distributions in H and I modes from the model. The dwell-time distributions from the model for 𝓒 = 1*μ*M and 𝓘 = 100nM in the H (Fig 5A–5D) and I (Fig 5E–5H) modes calculated by using Eqs (18) and (19) and parameters in Tables 1 and 3 are given by red lines. The experimental data are presented by the gray bars for comparison. A close inspection of the modal open and closed dwell-time distributions of IP_3_R_PS1WT_ and IP_3_R_PS1M146L_ provides useful insight into the modal behavior of the channel. In line with the over-all open dwell-time distribution, the open dwell-time distributions in the H (Fig 5A, 5C) and I (Fig 5E, 5F) modes do not change significantly. The closed dwell-time distributions in the two modes in IP_3_R_PS1M146L_ (Fig 5D, 5H) on the other hand, shift significantly to the left as compared to IP_3_R_PS1WT_ (Fig 5B, 5G). Furthermore, the shift in the closed dwell-time distribution in the H mode is more significant (Fig 5B, 5D). This suggests that the relatively shorter time spent by IP_3_R_PS1M146L_ in the H mode’s closed state plays a major role in the shortening of *τ*_c_ and hence enhancement of *P*_o_ of the channel in the presence of FAD-causing mutation as compared to wild-type PS1.

**Fig 6:**
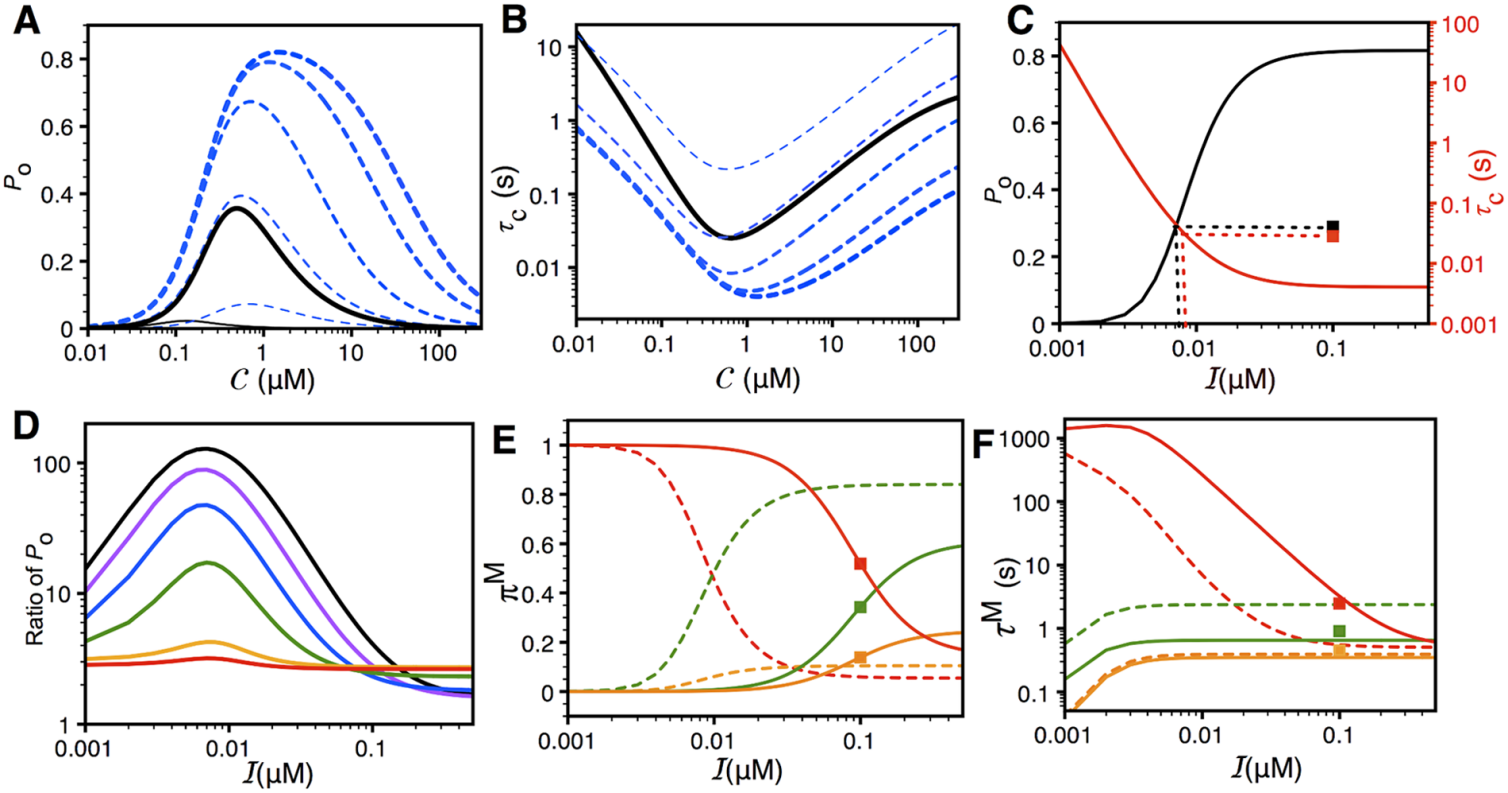
Quantifying the sensitization of IP_3_R in the presence of FAD mutant through simulations. The lines are from the model while the squares in panels (C, E, and F) are the experimental values for IP_3_R_PS1WT_ at 𝓒 = 1*μ*M and 𝓘 = 100nM. The increasing thicknesses of the lines in (A) and (B) represent increasing IP_3_ concentration. (A) *P*_o_ of IP_3_R_PS1WT_ at 𝓘 = 8nM and 100nM (black solid lines) and IP_3_R_PS1M146L_ at 𝓘 = 4nM, 8nM, 16nM, 40nM, and 100nM (blue dashed lines) as a function of 𝓒. (B) The *τ*_c_ of the channel from simulations in (A) where the thicknesses, colors, and styles of the lines have the same meanings as in (A), *τ*_c_ of IP_3_R_PS1WT_ at 𝓘 = 8nM is not shown. (C) The *P*_o_ (black) and *τ*_c_ (red) of IP_3_R_PS1M146L_ as a function of 𝓘 at 𝓒 = 1*μ*M. The vertical and horizontal black and red dotted lines respectively represent the 𝓘 value (∼ 8nM) at which the *P*_o_ and *τ*_c_ of IP_3_R_PS1M146L_ cross the *P*_o_ and *τ*_c_ of IP_3_R_PS1WT_ at 𝓒 = 1*μ*M and 𝓘 = 100nM. (D) The ratio of the *P*_o_ of IP_3_R_PS1M146L_ to that of IP_3_R_PS1WT_ as a function of 𝓘 at 𝓒 = 70nM (red), 100nM (yellow), 250nM (green), 500nM (blue), 1*μ*M (purple), and 2*μ*M (black). (E) and (F) show the prevalences and mean life-times respectively of L (red), I (orange), and H (green) modes of IP_3_R_PS1WT_ (solid lines) and IP_3_R_PS1M146L_ (dashed lines) as a function of 𝓘 at 𝓒 = 1*μ*M. Squares represent values observed in [15].

To gain further insights and quantify the extent of IP_3_R sensitization due to PS1-M146L, we extrapolate the *P*_o_, *τ*_c_, and the mean modal properties of IP_3_R at different values of 𝓒 and 𝓘 from our model using parameters tabulated in Tables 1 and 3. Fig 6A shows *P*_o_ of IP_3_R_PS1WT_ (black solid lines) and IP_3_R_PS1M146L_ (blue dashed lines) as a function of 𝓒 at different 𝓘, calculated using Eq (2). Even at 𝓘 = 8nM, *P*_o_ of IP_3_R_PS1M146L_ is already higher than that of IP_3_R_PS1WT_ at 𝓘 = 100nM (thick solid black line) for all 𝓒. Thus, whereas IP_3_R_PS1WT_ in 8 nM IP_3_ are minimally active (*P*_o_ ∼ 0.005) in resting 𝓒 (∼ 70 nM), IP_3_R_PS1M146L_ under the same ligand conditions can have sufficient activity (*P*_o_ > 0.04) to initiate intracellular Ca^2+^ signals.

The *τ*_c_ of the channels from simulation Eq (7) in Fig 6B correlate well with the *P*_o_ values (notice that at fixed 𝓘, *P*_o_ decreases as *τ*_c_ increases and vice versa). This close correlation between the *P*_o_ and *τ*_c_ is the consequence of the lack of dependence of the *τ*_o_ on 𝓘 (see Eq (7) and Fig 3C). Thus for 𝓘 > 8 nM, *τ*_c_ of IP_3_R_PS1M146L_ (blue dashed lines) is shorter than that of IP_3_R_PS1WT_ at 𝓘 = 100nM (black solid line) for all physiological 𝓒 values. Whereas the strong Ca^2+^ activation of IP_3_R_PS1M146L_ between 𝓒 = 0.01 and 0.5 *μ*M remains the same as 𝓘 is raised from 16 to 100 nM, channel *P*_o_ for higher 𝓒 does increase as 𝓘 increases from 16 nM to 1 *μ*M, confirming the higher saturating *P*_o_ of IP_3_R_PS1M146L_ as described above (Fig 3A) and is in line with observations (Table S1 in [14]).

To quantitatively compare the sensitivity of IP_3_R_PS1WT_ and IP_3_R_PS1M146L_ to activation by IP3, we plot in Fig 6C the *P*_o_ of IP_3_R_PS1M146L_ as a function of 𝓘 at fixed 𝓒 = 1*μ*M (black line) and the experimentally observed *P*_o_ of IP_3_R_PS1WT_ at 𝓒 = 1*μ*M and 𝓘 = 100 nM (black square). The plot reveals that *P*_o_ of IP_3_R_PS1M146L_ at 𝓘 = 8 nM already exceeds that of IP_3_R_PS1WT_ at 𝓘 = 100 nM (see black dotted lines). In contrast, *P*_o_ of IP_3_R_PS1WT_ is negligible at 𝓘 = 8nM and 𝓒 = 1*μ*M (Fig 6A, black thin solid line). Correspondingly, *τ*_c_ of IP_3_R_PS1M146L_ (red line in Fig 6C) becomes shorter than the observed *τ*_c_ of IP_3_R_PS1WT_ (red square) at 𝓘 = 100nM as 𝓘 is raised beyond 8nM.

In Fig 6D, we show the ratio of simulated *P*_o_ of IP_3_R_PS1M146L_ to that of IP_3_R_PS1WT_ as a function of 𝓘 at 𝓒 = 70nM, 100nM, 250nM, 500nM, 1*μ*M, and 2*μ*M. For 𝓘 < 200nM, IP_3_R_PS1M146L_ is more than twice as active as IP_3_R_PS1WT_ for all 𝓘 and 𝓒 values. For physiological resting 𝓒 = 70nM, IP_3_R channel activity is enhanced by 265% in cells expressing PS1-M146L relative to that in PS1-WT expressing cells. For optimal 𝓒, IP_3_R_PS1M146L_ exhibits a gain-of-function enhancement by over 100 folds as compared to IP_3_R_PS1WT_, with maximum enhancement occurring around 𝓘 = 7–8 nM IP_3_. The drop in the *P*_o_ ratio for 𝓘 > 8nM is due to the fact that *P*_o_ of IP_3_R_PS1M146L_ peaks much faster than IP_3_R_PS1WT_ as a function of 𝓘.

At fixed 𝓒 = 1*μ*M, the theoretical *π* ^L^ Eq (3) of IP_3_R_PS1M146L_ decreases for 2nM < 𝓘 < 100nM and plateaus outside this window (Fig 6E, dashed red line). *π* ^H^ of IP_3_R_PS1M146L_ (dashed green line) changes in the opposite direction for 2nM < 𝓘 < 100nM. *π* ^I^ of IP_3_R_PS1M146L_ (dashed orange line), on the other hand, remains largely constant. Around 𝓘 = 8 nM, both the *π* ^L^ and *π* ^H^ curves of IP_3_R_PS1M146L_ crosses the observed *π* ^L^ (red square) and *π* ^H^ (green square) levels, respectively, of IP_3_R_PS1WT_ measured at 𝓘 = 100nM and 𝓒 = 1*μ*M. *π* ^I^ of IP_3_R_PS1M146L_ gets close to but does not exceed that of IP_3_R_PS1WT_ observed at 𝓘 = 100nM and 𝓒 = 1*μ*M (orange square). This indicates that the increase in *P*_o_ of IP_3_R in the presence of PS1-M146L is mainly due the switching of the channel from L to H mode. Interestingly, the 𝓘 value (∼ 10nM) where *π* ^L^ and *π* ^H^ cross each other is almost the same as where *P*_o_ reaches half (0.41) of its peak value (0.82) (see black line in Fig 6C). This is in line with the observations that mode switching is the major mechanism of ligand regulation of IP_3_R [21]. Our results confirm that the mechanism of ligand regulation of IP_3_R is mainly due to the switching of the channel between L and H modes with minimal contributions from I mode [21]. Furthermore, switching of IP_3_R_PS1M146L_ from L to H mode in comparison to IP_3_R_PS1WT_ translates into the gain-of-function enhancement of IP_3_R gating. Fig 6E also shows how theoretical values of the prevalences of the three gating modes for IP_3_R_PS1WT_ vary with 𝓘 at 𝓒 = 1*μ*M (solid lines).

Theoretical values of the mean life-times of I (orange) and H (green) mode calculated using Eq (9) at 𝓒 = 1*μ*M remain largely unchanged for all values of 𝓘 > 3nM (Fig 6F). In that range of 𝓘, *τ* ^H^ of IP_3_R_PS1M146L_ (dashed green line) is shorter than *τ* ^H^ of IP_3_R_PS1WT_ (solid green line), while *τ* ^I^ of IP_3_R_PS1M146L_ (dashed orange line) remains almost the same as *τ* ^I^ of IP_3_R_PS1WT_ (solid orange line). For 𝓘 < 4nM, *τ* ^I^ of IP_3_R_PS1M146L_ drops below the observed value for IP_3_R_PS1WT_ at 𝓘 = 100nM. Simulated *τ* ^L^ of IP_3_R_PS1M146L_ (dashed red line), on the other hand, decreases significantly as 𝓘 increases. As 𝓘 increases beyond 15 nM, simulated *τ* ^L^ of IP_3_R_PS1M146L_ becomes shorter than that of IP_3_R_PS1WT_ measured at 𝓘 = 100nM (red square). Relatively speaking, this does not correlate very closely with the 𝓘 = 8nM where the *P*_o_ of IP_3_R_PS1M146L_ crosses the observed *P*_o_ of IP_3_R_PS1WT_ (Fig 6A) when compared to the prevalences where this critical value of 𝓘 is 8nM. Nevertheless, the shorter *τ* ^L^ will increase the *P*_o_ of IP_3_R_PS1M146L_. Thus the shorter *τ* ^L^ and longer *τ* ^H^ contribute to its increased sensitivity to IP_3_ as compared to IP_3_R_PS1WT_.

**Fig 7:**
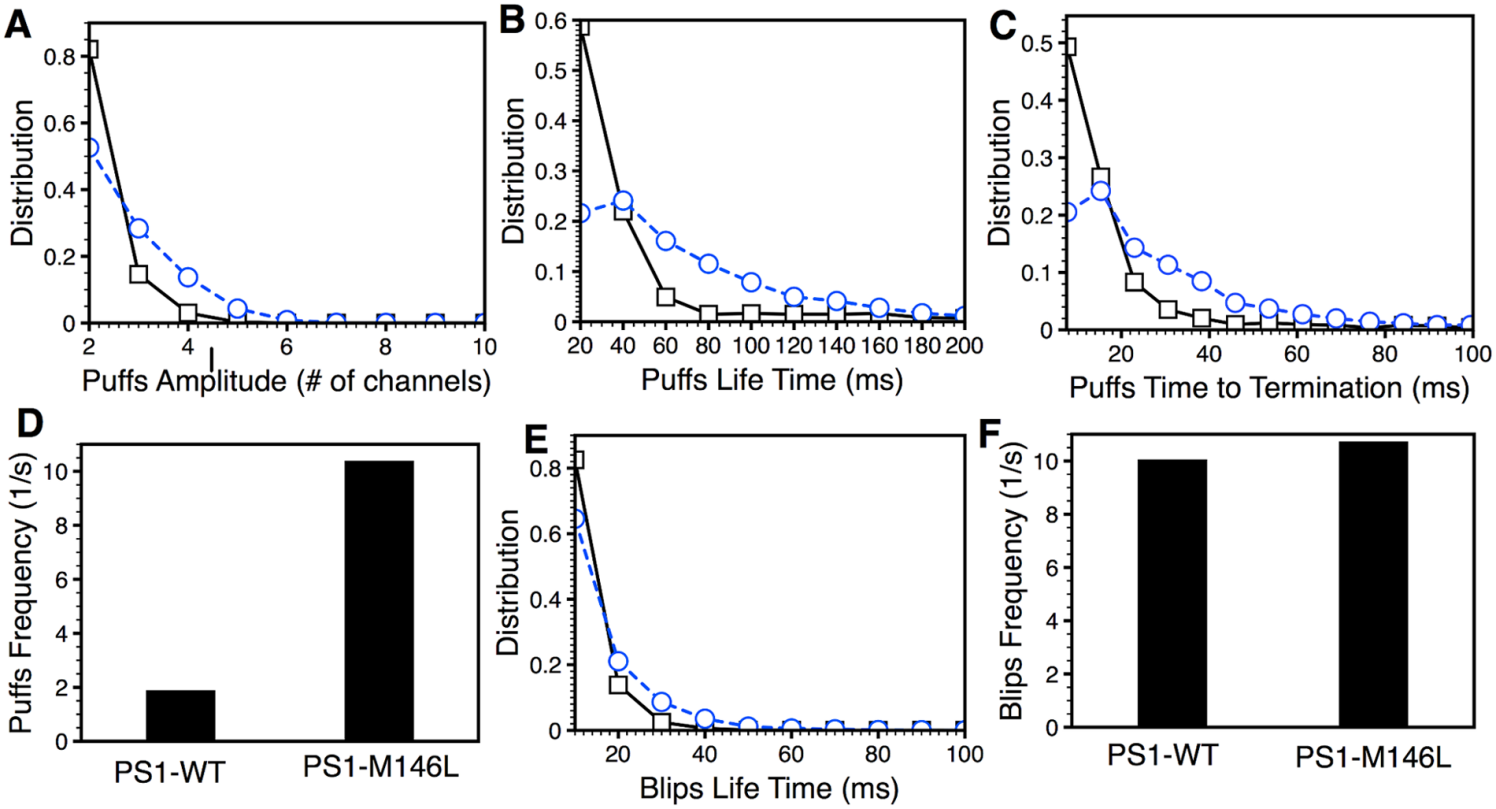
The statistics of Ca^2+^ puffs and blips change significantly in the presence of PS1-M146L (dashed lines with circles) as compared to PS1-WT (solid lines with squares). (A) Distribution of puff peak amplitude, (B) life time (duration from the start of the puff to the end of the puff), and (C) termination time (duration from the time at which maximum number of channels are open to the time when the last active channel becomes inactive). (D) Life time distribution of blips. Frequency of puffs (E) and blips (F). Simulations were performed at 100nM IP_3_ concentration.

To investigate how the remodeling of single IP_3_R channel gating kinetics in the presence of mutant PS1-M146L affects the dynamics of IP_3_R-mediated Ca^2+^ release events, we simulated such events at a Ca^2+^-release site consisting of a cluster of ten IP_3_Rs as described in the Methods Section. 400 s-long records of Ca^2+^ blips and puffs (Ca^2+^-release events involving just one, or multiple IP_3_R channels in the cluster, respectively) from the IP_3_R cluster were generated, and statistics about these Ca^2+^-release events were derived as described previously [28]. In line with observations reported in [35, 12], the site produces significantly potentiated puffs in the presence of FAD-causing PS1. As shown in Fig 7, the behavior of puffs and blips arising from a cluster of IP_3_R_PS1M146L_ (dashed lines with circles) is significantly different from that of a IP_3_R_PS1WT_ cluster (solid line with squares). Puffs from a IP_3_R_PS1M146L_ cluster have significantly larger amplitudes (Fig 7A), longer life times (Fig 7B), and take longer to terminate (Fig 7C). Furthermore, puff frequency in cells expressing PS1-M146L is significantly higher than that in PS1-WT-expressing cells (10.33/sec versus 1.83/sec) (Fig 7D). Interestingly, the statistics of Ca^2+^ blips are very similar in both case. Although the life times of blips in PS1-M146L-expressing cells is slightly longer than that in PS1-WT-expressing cells (Fig 7E), frequencies of the blips are almost the same (10.67/sec versus 9.99/sec) (Fig 7F). Thus the higher number of single-channel events caused by higher sensitivity of IP_3_R_PS1M146L_ to activation by IP_3_ translates into triggering more frequent and longer puffs.

## Discussion

Accumulation of A*β* aggregates and intracellular neurofibrillary tangles of *τ* protein are the main symptoms of AD [1]. However, most drugs focused on restricting A*β* production and accumulation or enhancing its clearance from the brain have yielded disappointing results [36]. This could be due to the fact that these drugs target the late stage features of the disease, i.e. plaque formation—whereas there is poor correlation between A*β* deposits and the progressive memory loss and cognitive decline observed [37]. Compelling evidence suggests that FAD-causing mutant PS disrupt intracellular Ca^2+^ signaling before A*β* deposition, pointing towards the up-regulated Ca^2+^ signaling as a proximal event that could be involved in disease pathogenesis. Previously, we showed that FAD-causing mutant PS interact with ER-localized IP_3_Rs, leading to their gain-of-function enhanced channel activity [14, 15]. However, further analysis is needed to elucidate the modified gating properties leading to the gain-of-function enhancement of IP_3_R channel activity and to quantify their sensitization due to FAD-causing PS mutations.

Our modeling results show that there is a significant increase in the H mode prevalence, and a corresponding significant reduction in the L mode prevalence of IP_3_R channels in the presence of PS1-M146L. This change in the prevalence of L and H modes arises mainly from the shorter mean life-time of the L mode and longer mean-life-time of the H mode. On the other hand, both the life-time and prevalence of I mode remain constant. Our model predicts that the *P*_o_ of IP_3_R_PS1M146L_ saturates at significantly lower IP_3_ concentrations and its peak value is higher compared to that of IP_3_R_PS1WT_. Furthermore, our model predicts that the channel’s gain-of-function enhancement is sensitive to both IP_3_ and Ca^2+^ (Fig 6A). Interestingly, our model predicts that the higher *P*_o_ of IP_3_R_PS1M146L_ as compared to IP_3_R_PS1WT_ is mainly due to the lower occupancy of ${\text{C}}_{32}^{\text{L}}$ state and higher occupancy of the ${\text{O}}_{24}^{\text{H}}$ state (with small change in the occupancies of ${\text{O}}_{24}^{\text{I}}$ and ${\text{O}}_{14}^{\text{I}}$). This means that PS1-M146L interacts with IP_3_R in such a way that the closed configuration of the channel with 3 Ca^2+^ ions and 2 IP_3_ bound is less likely and the open configuration in the H mode with 2 Ca^2+^ ions and 4 IP_3_ molecules bound more easily attainable. The changes in the occupancies of states ${\text{O}}_{24}^{\text{H}}$ and ${\text{C}}_{32}^{\text{L}}$ lead to the increase in the prevalence of H mode and the corresponding decrease in the prevalence of L mode, resulting in the gain-of-function enhancement of IP_3_R gating in the presence of PS1-M146L.

Our goal in this study was to use all the data at our disposal to model the gating kinetics of IP_3_R_PS1WT_ and IP_3_R_PS1M146L_; and use resulting models to simulate the behavior of IP_3_R_PS1WT_ and IP_3_R_PS1M146L_ in a wide range of ligand concentrations to gain a better understanding of how the gain-of-function enhancement of IP_3_R_PS1M146L_ alters the characteristics of local Ca^2+^ release events at IP_3_R clusters.

Because of the considerable technical difficulties in obtaining single-channel current records of IP_3_R channels in their native membrane milieu by nuclear patch clamp electrophysiology, the most comprehensive set of such single-channel data (including steady-state gating records over a broad range of combinations of cytoplasmic IP_3_ and Ca^2+^ [23], long gating records in multiple constant ligand conditions suitable for modal gating analysis [21], records of response kinetics of IP_3_R channel to rapid changes in cytoplasmic ligand conditions [38]) was obtained in the study of the endogenous IP_3_R from insect Sf9 cells. This set of data was used to develop the twelve-state kinetic model [22] that provides the basis of the models we develop here to simulate the behavior of the endogenous Sf9 IP_3_R in the presence of exogenous recombinant human PS1 (WT and mutant). Until a comparable or more comprehensive set of single-channel data for a mammalian IP_3_R becomes available, our approach is the best that can be achieved to simulate gating behaviors of IP_3_R interacting with WT and mutant PS1. Although Sf9 IP_3_R does not interact with human PS1 in its natural environment, the study in [15] showed that endogenous IP_3_R in human lymphoblasts in presence of PS1-M146L exhibits very similar changes in its gating characteristics (increase in *P*_o_ and *τ*_o_ with corresponding reduction in *τ*_c_) and modal gating behavior (rise in *π* ^H^ with simultaneous drop in *π* ^L^) when compared to IP_3_R in the presence of PS1-WT. Therefore, we have reason to be confident that simulation results from our modeling effort do reflect the gating behaviors of IP_3_R naturally interacting with WT and mutant PS1, and that the insights our effort provides about the effects of mutant PS1 on IP_3_R single-channel gating and local intracellular Ca^2+^ release events can improve our understanding of the pathophysiology of FAD.

The higher prevalence and longer life-time of H mode and shorter life-time of L mode for IP_3_R_PS1M146L_ may have important implications for cell physiology. Since the open time in the H mode is significantly higher than that in the L mode, in which the channel has near-zero mean open time, IP_3_R gating in the H mode will have higher probability of activating neighboring channels through Ca^2+^-induced-Ca^2+^ release (CICR), thus leading to higher frequency of Ca^2+^ puffs (Fig 7D). The higher sensitivity of IP_3_R_PS1M146L_ to activation by Ca^2+^ and IP_3_ also increases the number of open channels in an IP_3_R channel cluster during a puff, leading to bigger (Fig 7A) and longer (Fig 7B) puffs, which in turn will cause more frequent global Ca^2+^ waves and oscillations at the cellular level, in line with observations [35, 12]. The higher prevalence of IP_3_R channel being in the H mode resulting in the higher probability of inducing global Ca^2+^ events can account for the higher frequency of Ca^2+^ oscillations in B lymphoblasts from FAD patients and DT40 cells expressing FAD-causing mutant PS [14, 15].

Our model reveals that even at resting level of 𝓘 (8 nM), IP_3_R_PS1M146L_ exhibits significant *P*_o_ of 0.35 (40% of the *P*_o_ when the channel is in saturating 𝓘) at 𝓒 = 1*μ*M whereas the *P*_o_ of IP_3_R_PS1WT_ in the same ligand condition is negligible. This will lead to stronger CICR among channels in the same cluster and between IP_3_R channel clusters, thereby generating more global spontaneous Ca^2+^ oscillations in cells expressing FAD-causing mutant PS as observed experimentally [14, 15].

To conclude, our study provides insights into the gating modulation of IP_3_R that leads to the gain-of-function enhancement due to FAD-causing mutations in PS. Furthermore, significant activity exhibited by IP_3_R at resting IP_3_ concentration in cells expressing FAD-causing mutant can explain the spontaneous global Ca^2+^ signals observed in those cells. The models developed here for single-channel IP_3_R channel gating and local Ca^2+^-release events can provide part of the foundation for building whole-cell models to judiciously separate the key pathways leading to the global Ca^2+^ signaling dysregulation in AD from those that are by-products due to the CICR nature of Ca^2+^ signaling. For example, what are the relative contributions of gain-of-function enhancement and over-expression of ryanodine receptors [39, 20] to Ca^2+^ signaling dysregulation in AD? Does the down-regulation of Ca^2+^ buffers such as calbindin [40, 41] and higher resting Ca^2+^ concentration [42] play a role (through CICR mechanism) in the exaggerated Ca^2+^ signals? What are the conditions or factors that would reverse the exaggerated Ca^2+^ signaling back to normal state? These and many other interesting questions are the focus of our future research and the models developed here will play a key role in addressing them.

## Supporting Information

S1 Text Stochastic scheme and diffusion of Ca^2+^ and dye buffer. (PDF)
