# Supplementary material for: Analyzing and Quantifying the Gain-of-Function Enhancement of IP3 Receptor Gating by Familial Alzheimer’s Disease-Causing Mutants in Presenilins
Source: PLoS Comput Biol. 2015 Oct 6;11(10):e1004529. doi: 10.1371/journal.pcbi.1004529 (PMC4595473; doi:10.1371/journal.pcbi.1004529)
Supplement: S1 Text — (PDF) [file pcbi.1004529.s001.pdf]

Supplemental Information Text: Analyzing and Quantifying the Gain-of-Function Enhancement of IP_3 Receptor Gating by Familial Alzheimer's Disease-Causing mutants in Presenilins

Don-On Daniel Mak[1], King-Ho Cheung[2], Patrick Toglia[3], J. Kevin Foskett[1,4], and Ghanim Ullah[3,*]
[1]Department of Physiology, University of Pennsylvania, Philadelphia, PA 19104, USA
[2]Department of Physiology, The University of Hong Kong, Pok Fu Lam, Hong Kong
[3]Department of Physics, University of South Florida, Tampa, FL 33620, USA,
[4]Department of Cell and Developmental Biology, University of Pennsylvania, Philadelphia, PA 19104, USA

1 Stochastic Scheme of Channel Gating

The gating of IP_3R is given by the 12-state model described in the main text. To determine the state of the channel, we have to determine the transition probabilities at a given time [1, 2]. That is, if the jth channel is in state i, we have to determine the probabilities with which it remains in that state or switches into another state allowed by the kinetic scheme shown in Fig. 1 (main text) within the time interval Δt. For example, if a channel is in state $\mathrm{C}_{00}^{\mathrm{L}}$, possible transitions are to states $\mathrm{C}_{20}^{\mathrm{L}}$ and $\mathrm{C}_{04}^{\mathrm{I}}$. For a sufficiently small time interval Δt, the probabilities for these transitions are given by $P^{(j)}_{\mathrm{C}_{00}^{\mathrm{L}}\to\mathrm{C}_{20}^{\mathrm{L}}} = r_{(\mathrm{C}_{00}^{\mathrm{L}}\to\ \mathrm{C}_{20}^{\mathrm{L}})}\Delta t$ and $P^{(j)}_{\mathrm{C}_{00}^{\mathrm{L}}\to\mathrm{C}_{04}^{\mathrm{I}}} = r_{(\mathrm{C}_{00}^{\mathrm{L}}\to\ \mathrm{C}_{04}^{\mathrm{I}})}\Delta t$. The probability for the channel to remain in state $\mathrm{C}_{00}^{\mathrm{L}}$ is $P_{\mathrm{C}_{00}^{\mathrm{L}}\to\mathrm{C}_{00}^{\mathrm{L}}} = 1 - P_{\mathrm{C}_{00}^{\mathrm{L}}\to\mathrm{C}_{20}^{\mathrm{L}}} - P_{\mathrm{C}_{00}^{\mathrm{L}}\to\mathrm{C}_{04}^{\mathrm{I}}}$. To determine the transition probabilities, we divide the unit interval into three subintervals of length $P_{\mathrm{C}_{00}^{\mathrm{L}}\to\mathrm{i}}\Delta t$, i represent the three states to which the channel can make transition. If a random number drawn from a uniform distribution over the unit interval falls into the subinterval $P_{\mathrm{C}_{00}^{\mathrm{L}}\to\mathrm{i}}\Delta t$, the corresponding transition is

performed. The time interval Δt was kept small enough for the linear dependence of $P_{i\to i}$ on the time interval to remain valid. We used a time step of 1 μs for the puff simulations. The channel is open when in any of the states $\mathrm{O}_{14}^{\mathrm{I}}$, $\mathrm{O}_{24}^{\mathrm{I}}$, or $\mathrm{O}_{24}^{\mathrm{H}}$. The above procedure was repeated for all channels.

2 Elements of Tridiagonal Matrix (TM)

Considering a spherical symmetry around the channel, the system of eqs. (26, main text) and (27, main text) converts to a TM system. Here we derive the elements of the TM. The detail derivation of the scheme is given in [3]. This method converts a 3D problem into a 1D problem and is numerically significantly faster than the standard methods such as Crank-Nicolson. In what follows n is the time index and j is the space index in spherical polar coordinates. Using eq. (29, main text), we can write eq. (26, main text) as

$$\frac{c^{(n+1)} - c^{(n)}}{\Delta t} = D\nabla^2 c^{(n+1)} + J\delta(r) + k_d^r(B_d - b_d^{(n)}) - k_d^f c^{(n+1)} b_d^{(n)} \tag{1S}$$

Writing the Laplacian in spherical polar coordinates and considering no-flux boundary conditions we get the elements of lower, middle, and upper diagonal (a_j, b_j, and c_j respectively) of the TM given as

$$a_j = \begin{cases} 0 & \text{if } j = 1 \\ \frac{-D\Delta t}{\Delta r^2}(1 - \frac{1}{2j})^2 & \text{if } j = 2, ...N - 1 \\ \frac{-2D\Delta t}{\Delta t} & \text{if } j = N. \end{cases} \tag{2S}$$

$$b_j = \begin{cases} \frac{6D\Delta t}{\Delta r^2} + 1 + k_d^f b_d^{(n,j)} \Delta t & \text{if } j = 1 \\ \frac{D\Delta t}{\Delta r^2}(1 + \frac{1}{2j})^2 + \frac{D\Delta t}{\Delta r^2}(1 - \frac{1}{2j})^2 + 1 + k_d^f b_d^{(n,j)} \Delta t & \text{if } j = 2, ..N - 1 \\ \frac{2D\Delta t}{\Delta r^2} + 1 + k_d^f b_d^{(n,j)} \Delta t & \text{if } j = N. \end{cases} \tag{3S}$$

$$c_j = \begin{cases} \frac{-6D\Delta t}{\Delta r^2} & \text{if } j = 1 \\ \frac{-D\Delta t}{\Delta r^2}(1 + \frac{1}{2j})^2 & \text{if } j = 2, ...N - 1 \\ 0 & \text{if } j = N. \end{cases} \tag{4S}$$

The right hand side for the TM system for the cytosolic Ca^{2+} concentration is represented by d_j and is

$$d_j = c^{(n,j)} + J\delta(r)\Delta t + k_d^r(B_d - b_d^{(n,j)})\Delta t, \text{ j=1,..N} \tag{5S}$$

The rate equation for dye buffer can be expanded and solved iteratively in similar fashion.

References

1. Ullah G, Jung P (2006) Modeling the statistics of elementary calcium release events. Biophysical Journal 90: 3485–3495.

2. Swaminathan D, Ullah G, Jung P (2009) A simple sequential-binding model for calcium puffs. Chaos 19: 037109.

3. Ullah G, Parker I, Mak DOD, Pearson JE (2012) Multi-scale data-driven modeling and observation of calcium puffs. Cell calcium 52: 152–160.

4. Falcke M (2004) Reading the patterns in living cellsthe physics of Ca 2+ signaling. Advances in physics 53: 255–440.

5. Smith IF, Parker I (2009) Imaging the quantal substructure of single IP3R channel activity during Ca2+ puffs in intact mammalian cells. Proceedings of the National Academy of Sciences 106: 6404-6409.

6. Williams A, West D, Sitsapesan R (2001) Light at the end of the Ca2+-release channel tunnel: structures and mechanisms involved in ion translocation in ryanodine receptor channels. Quarterly Reviews of Biophysics 34: 61–104.

7. Taylor C, da Fonseca P, Morris E (2004) IP3 receptors: the search for structure. Trends in biochemical sciences 29: 210–219.

8. Rüdiger S, Shuai JW, Sokolov IM (2010) Law of mass action, detailed balance, and the modeling of calcium puffs. Phys Rev Lett 105: 048103.

9. Allbritton N, Meyer T, Stryer L (1990) Range of messenger action of calcium ion and inositol 1, 4, 5-trisphosphate. Genes Dev 4: 1753.

10. Dargan S, Parker I (2003) Buffer kinetics shape the spatiotemporal patterns of IP3-evoked Ca2+ signals. The Journal of Physiology 553: 775.

11. Shuai J, Parker I (2005) Optical single-channel recording by imaging Ca2+ flux through individual ion channels: theoretical considerations and limits to resolution. Cell Calcium 37: 283–299.

Table 1S: Parameters for puff simulations

Quantity	Symbol	—	Numerical Value	Reference
Resting Cytosolic Calcium	Ca_{rest}	=	$50nM$	[4]
Dye Buffer	B_d	=	$20\ \mu M$	
Number of channels	N_{ch}	=	10	[5]
Pore Radius	r_{pore}	=	$2.5nm$	[6, 7]
Channel Spacing	r_{nn}	=	$120nm$	[3, 8]
Ca^{2+}	D_c	=	$0.223\mu m^2/ms$	[9]
Dye	D_d	=	$0.200\mu m^2/ms$	[10]
Dye Buffer	k_d^f	=	$0.15/\mu Mms$	[10, 11]
	k_d^r	=	$0.45/ms$	[10, 11]
